# Skillful Introduction of Urea during the Synthesis of MOF-Derived FeCoNi–CH/p-rGO with a Spindle-Shaped Substrate for Hybrid Supercapacitors

**DOI:** 10.1021/acsomega.2c02712

**Published:** 2022-09-06

**Authors:** Yu Zhang, Chen-Ming Liang, Min Lu, Hao Yu, Guang-Sheng Wang

**Affiliations:** † School of Chemical Engineering, Northeast Electric Power University, Jilin 132000, China; ‡ School of Chemistry, Beihang University, Beijing 100191, China

## Abstract

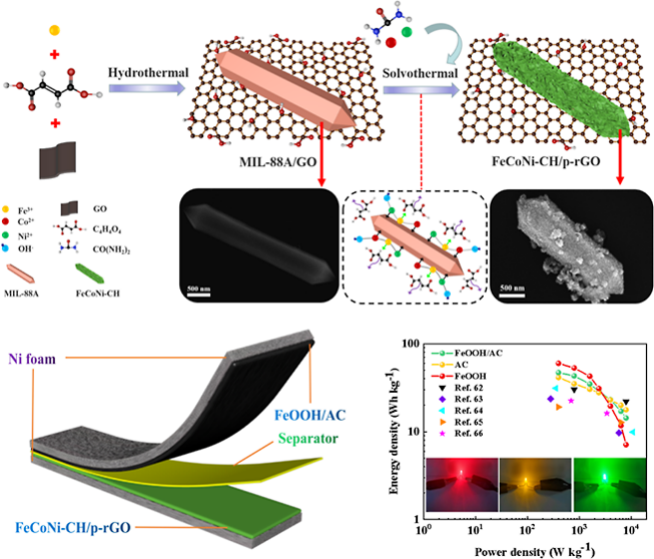

A composite (FeCoNi–CH/p-rGO) with a spindle-shaped substrate is controllably prepared by combining FeCoNi carbonate hydroxide (FeCoNi–CH) and partially reduced graphite oxide (p-rGO) using a novel chemical strategy. In the synthetic process, urea is introduced as the precipitant and reducing agent. MIL-88A as a self-template is converted into a ternary-metal CH composite, maintaining the original morphology by the metal ion etching and coprecipitation method, and graphite oxide is reduced to rGO with stronger conductivity partially at the same time. The electrochemical performance of the FeCoNi–CH/p-rGO is superior to FeCoNi–CH, with a high specific capacitance (1346 F g^–1^ at 0.5 A g^–1^) and rate capability (55.5% at 10 A g^–1^). The better electrochemical performance of the FeCoNi–CH/p-rGO composite is attributed to the pseudocapacitive energy storage capacity caused by the synergistic action of ternary-metal CH and the high conductivity of p-rGO. Meanwhile, the uniform mixture of FeOOH/activated carbon (AC) is fabricated as an anode to instead of the pure FeOOH or AC, which leads to the balancing energy density and high cycle stability of the hybrid supercapacitor (HSC). The corresponding assembled FeCoNi–CH/p-rGO//FeOOH/AC HSC exhibits a high energy density of 46.93 W h kg^–1^ at 400 W kg^–1^ power density and a cycle stability of 66.7% after 3000 cycles. In addition, this work also provides a facile method to fabricate metal–organic framework-derived ternary-metal CH/p-rGO composite materials, which could be applied in the fields of supercapacitors and other fields.

## 1 Introduction

The supercapacitors have been widely used in rail transit, electric buses, wind power, smart grid, and other emerging markets due to its excellent characteristics of high power density, quick charge and discharge, long cycle life, safety and reliability, and environmental friendliness.^1^ Supercapacitors can be classified into electric double-layer capacitors (EDLCs), pseudocapacitors (PCs), and hybrid capacitors (HSCs) according to the charge storage mechanism of electrode materials. The electrode material, one of the components of the supercapacitors, has great effects on charge storage. Hence, the design and development of high-performance electrode materials has become one of the research hotpots in the field of supercapacitors in recent years.^2,3^ The electrode materials used in EDLCs are mainly carbonaceous materials (for instance, graphene, activated carbon, etc.), but the whole bulk of the material is not fully utilized, which results in low and unsatisfactory energy density. Transition metal oxides,^4^ hydroxides,^5^ sulfides,^6^ phosphating compounds,^7^ and conductive polymers^8^ are common typical electrode materials of PCs. Meanwhile, HSCs consist of a EDLC anode and PC cathode with their synergy effect, which is conducive to the improvement of the energy density.^9^ Among many electrode materials, the transition metal carbonate hydroxide (CH) has become a potential pseudocapacitive electrode material because of its metal ion tunability and a large number of redox active sites.^10^ At present, these results are mainly focused on mono-metal or binary-metal CH, such as Co–CH,^11^ Zn–CH,^12^ NiCo–CH,^13^ NiCu–CH,^14^ NiFe–CH,^15^ CoFe–CH,^16^ and so forth. However, on account of the introduction of a third metal elements, the rarely reported ternary-metal CH (such as NiCoMn–CH^17^ and ZnNiCo–CH^18^) should have a synergistic effect to raise the conductivity of the materials and provide more redox active sites.^17^ These previously reported M–CH was frequently prepared by the one-pot method, resulting in a single material shape, poor adjustability, and easy lamellae aggregation.

Furthermore, the morphology of electrode materials affects the electrochemical performance of composites significantly.^19^ Metal ion etching using metal–organic framework (MOF) materials with large specific surface area and pore size as well as high porosity can be acted as self-templates,^20^ resulting in CH nanosheets distributed on the templates with various surface morphologies. However, the bottleneck problems of CH composites, such as poor conductivity and cycling stability, seriously restrict their further application. Therefore, it is necessary to a design novel CH-based composite with a combination of other special forms and other materials such as carbon nanotubes,^21^ carbon fiber,^22^ carbon cloth,^23^ graphene,^24^ and other carbon materials with good electrical conductivity. Graphene is the most widely used of these carbon materials. GO is commonly used as a precursor in graphene composites, which is subsequently chemically reduced to reduced graphene oxide (rGO). Chemical reductants such as N_2_H_4_·H_2_O, NaBH_4_, and HI are commonly utilized.^25−27^

Herein, a novel chemical strategy was used to prepare a ternary-metal composite (FeCoNi–CH/p-rGO) using MIL-88A as a self-template and urea as a precipitant and reducing agent. The MIL-88A template’s original peculiar shape was kept, preventing lamellae aggregation. Meanwhile, it solved the problem of adding extra harmful reducing chemicals (such as N_2_H_4_·H_2_O and NaBH_4_) to traditional reduction procedures. As a result, the as-prepared FeCoNi–CH/p-rGO composite exhibited high specific capacitance (1346 F g^–1^ at 0.5 A g^–1^) and rate capability (55.5% at 10 A g^–1^). In addition, in order to obtain better electrochemical performance, we chose a FeOOH/activated carbon (AC) (*m*_FeOOH_/*m*_AC_ = 1:2) mixture to substitute pure FeOOH or AC as the anode, which combines EDLC and PC charge storage mechanisms. The related self-assembling FeCoNi–CH/p-rGO//FeOOH/AC HSC displayed a high energy density of 46.93 W h kg^–1^ at 400 W kg^–1^ and cycle stability of 66.7% after 3000 cycles.

## 2 Experimental Section

### 2.1 Materials

Graphite oxide (GO) dispersion was bought from Jiangsu Hengqiu Technology Co., Ltd. (Jiangsu Province, China). All the other chemical reagents used in this experiment were analytically pure without further purification process.

### 2.2 Preparation of MIL-88A and MIL-88A/GO

MIL-88A was prepared according to the previous preparation protocol.^28^ MIL-88A/GO was prepared by a hydrothermal method, which involved the following processes. First, a certain amount of graphite oxide at a concentration of 5 mg/mL (0.25, 0.5, 1, or 2 mL) was dispersed by ultrasound in 15 mL of ultrapure water for 15 min, and the resulting solution was stirred magnetically for 30 min. According to the calculation of the relative molecular weight of the MIL-88A monomer, the contents of 0.25, 0.5, 1, and 2 mL GO accounted for approximately 0.25, 0.5, 1, and 2 wt %, respectively. Second, 3 mmol (810 mg) FeCl_3_·6H_2_O and 3 mmol (348 mg) fumaric acid were successively added into the solution and vigorously stirred for 30 min and 1 h, respectively. Then, it was transferred to a 30 mL Teflon-lined stainless steel reactor and placed in the oven to react at 65 °C for 12 h. After cooling to room temperature, the products were washed by centrifuging with ultrapure water and anhydrous ethanol three times and dried in a vacuum oven at 60 °C overnight.

### 2.3 Fabrication of FeCoNi–CH and FeCoNi–CH/p-rGO

FeCoNi–CH was fabricated by a self-template method. First, 22 mg of MIL-88A was dissolved in 12 mL of anhydrous ethanol and stirred magnetically for 10 min to obtain solution A. Moreover, different ratios of Co(NO_3_)_2_·6H_2_O and Ni(NO_3_)_2_·6H_2_O (300:0/200:100/150:150/100:200/0:300 mg) as well as 200 mg of urea were dissolved in 8 mL of ultrapure water and the mixture was stirred for 10 min to obtain solution B. Then, solution A and solution B were mixed and stirred for 30 min and transferred to a 100 mL Teflon-lined stainless steel reactor for 5.0 h at 90 °C. When cooled to room temperature, the samples were dried overnight in a 60 °C vacuum oven after regular washing by centrifugation to obtain FeCoNi–CH with different molar ratios of Co/Ni. The fabrication process of FeCoNi–CH with different etching times was similar to that of FeCoNi–CH with different molar ratios of Co/Ni, with the difference being 150 mg of Co(NO_3_)_2_·6H_2_O as well as Ni(NO_3_)_2_·6H_2_O and a reacting time of 2.5/5.0/7.5/10.0/12.5 h, respectively. The fabrication process of FeCoNi–CH/p-rGO was similar to that of FeCoNi–CH with different etching time, except that MIL-88A was replaced by MIL-88A/GO with different GO contents, and the reaction time was 10 h.

### 2.4 Synthesis of FeOOH

FeOOH was synthesized by a hydrothermal strategy previously reported.^29^

### 2.5 Material Characterization

X-ray diffraction (XRD) diagrams were obtained using a X’Pert PRO MPD X-ray diffractometer with Cu Kα radiation (λ = 1.5406 Å) at a scan speed of 2° min^–1^. The morphology and size of the products were examined by scanning electron microscopy (SEM, Hitachi S4800). The element contents and chemical composition of the products were confirmed by energy-dispersive X-ray spectrometry (EDS) and X-ray photoelectron spectroscopy (XPS, Thermo ESCALAB 250). All XPS spectra were corrected using the C 1s line at 284.6 eV. Raman spectra were recorded using a Horiba Scientific LabRAM HR Evolution with a 532 nm laser source. The micromorphology and structure were further characterized by transmission electron microscopy (TEM, TECNAI F20). Fourier transform infrared (FT-IR) spectra were recorded on an IRAffinity-1 spectrometer with KBr pellets. The N_2_ adsorption and desorption isotherms were measured using a Micromeritics TriStar 3020 instrument in the static mode to get the results of specific surface area and pore structure.

### 2.6 Electrochemical Characterization and Tests

All electrochemical tests were achieved at room temperature with a three-electrode system using CHI 660E (Chenhua Instrument, Shanghai, China). The platinum plate and Hg/HgO electrode acted as counter electrodes and reference electrodes, respectively. The working electrode was fabricated by compounding the active substance, acetylene black, and polyvinylidene fluoride (PVDF) conductive adhesive uniformly at a mass ratio of 8:1:1 and pressing it into nickel foam (1 × 1 cm^2^) using a pressing machine. The nickel foam was then dried overnight in a vacuum oven at 60 °C. Furthermore, the electrochemical performance parameters of cyclic voltammetry (CV), galvanostatic charge–discharge (GCD), and electrochemical impedance spectroscopy (EIS) were tested in a 2 M KOH aqueous electrolyte to assess the electrochemical properties of electrode materials. The specific capacitance (*C*, F g^–1^) can be computed by the following equation
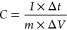
1where *I* (A), Δ*t* (s), *m* (g), and Δ*V* (V) denote discharge current, discharge time, mass of active substance on electrode, and potential window, respectively.

### 2.7 Fabrication of the Hybrid Supercapacitor

The mass ratio of the cathode and anode is ascertained on the basis of the charge storage balance rule by the following formula
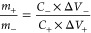
2where *m*_+_ and *m*_–_ are the loading mass (g); *C*_+_ and *C*_–_ are the specific capacitance (F g^–1^); and Δ*V*_+_ and Δ*V*_–_ are the voltage range (V); the plus and minus signs are the cathode and anode electrodes, respectively.

The electrochemical performance parameters of CV and GCD of HSC were measured in a 6 M KOH aqueous electrolyte. The energy density (*E*, W h kg^–1^) and power density (*P*, W kg^–1^) of the HSC are calculated according to the following equations
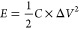
3
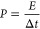
4where *C* (F g^–1^), Δ*V* (V), and Δ*t* (s) are electrochemical performance parameters of HSC, respectively.

## 3 Results and Discussion

### 3.1 Fabrication of the FeCoNi–CH/p-rGO Composite

**Scheme 1:**
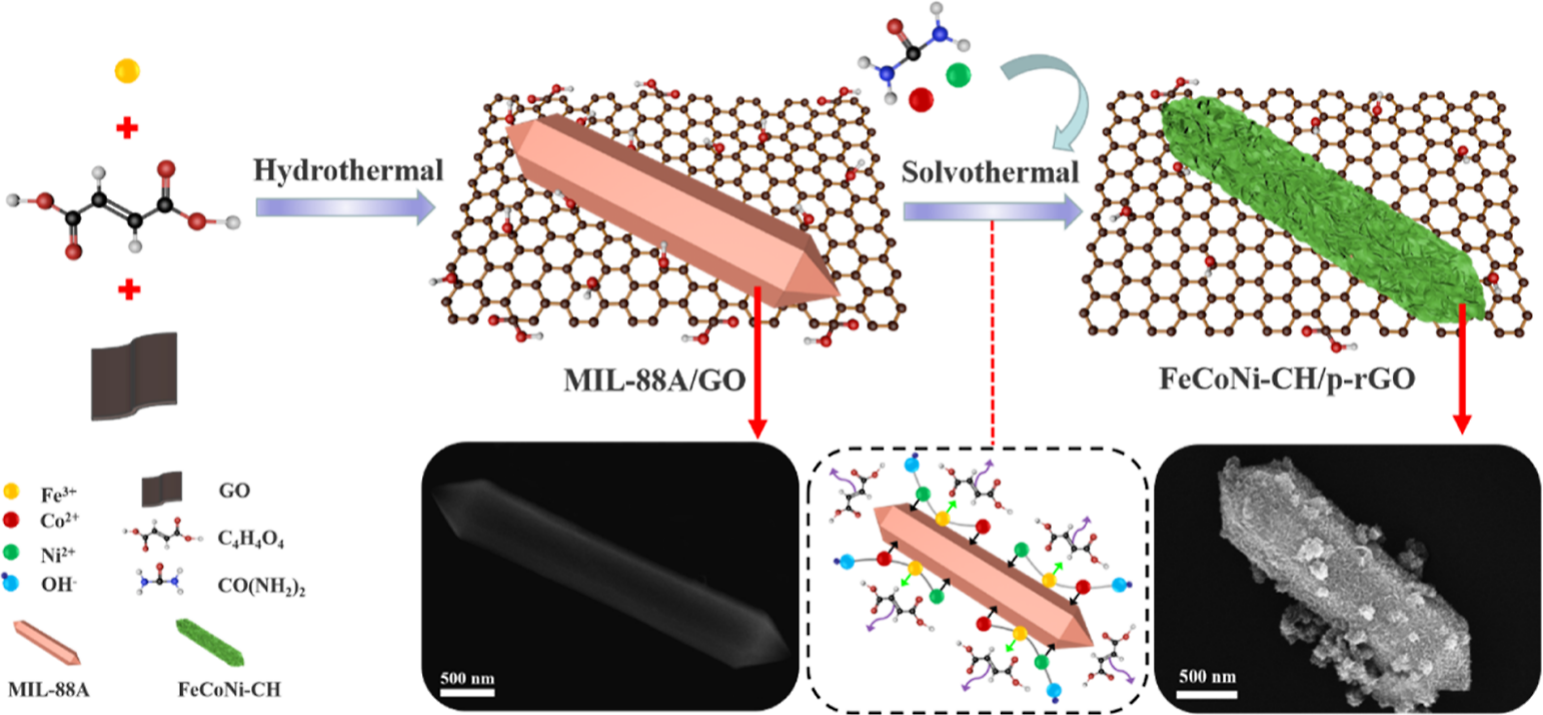
Diagram of the Preparation Process Mechanism of FeCoNi–CH/p-rGO

The possible synthetic mechanism of the FeCoNi–CH/p-rGO composites with a spindle-shaped substrate is shown in Scheme 1. First, the GO reacts with Fe^3+^ and fumaric acid to form MIL-88A/GO with the hydrothermal method. Moreover, the adscititious urea, Ni(NO_3_)_2_·6H_2_O, and Co(NO_3_)_2_·6H_2_O are mixed in the solution, and the sacrificial template MIL-88A is continuously etched during the hydrolysis reaction. When Fe^3+^ is released, FeCoNi–CH is formed by the coprecipitation of Fe^3+^, Ni^2+^, Co^2+^, OH^–^, and CO_3_^2–^ generated by the hydrolysis of urea as a precipitant. Meanwhile, urea can also work as the reducing agent to partially reduce GO to rGO, resulting in FeCoNi–CH/p-rGO composites. The reduction of GO is facilitated through the reducing species originated from the decomposition of urea.^30^ The underlying mechanism could be as follows: a prior investigation of GO reduction using NaOH or KOH reveals that an alkaline environment can promote the reduction of GO to rGO.^31^ It so happened that one of the hydrolysis products of urea, OH^–^, can provide an alkaline environment and it just so occurred. Furthermore, investigations have revealed that after reducing GO to rGO with urea, there are nitrogen-containing functional groups on rGO,^32^ which could be owing to the action of NH_4_^+^, another urea hydrolysis product, in the reduction process. The partial reduction of GO in this case is due to a shorter reaction period than the literature-recommended 30 h,^33^ so we can encourage GO reduction further by prolonging the reaction time or increasing the amount of urea used. During the synthetic process, the factors (Co/Ni ratios, etching and coprecipitation times, and the amount of GO) were accordingly regulated to optimize the electrochemical properties of the composite.

### 3.2 Structural, Morphological, and Composition Characterization

**Figure 1:**
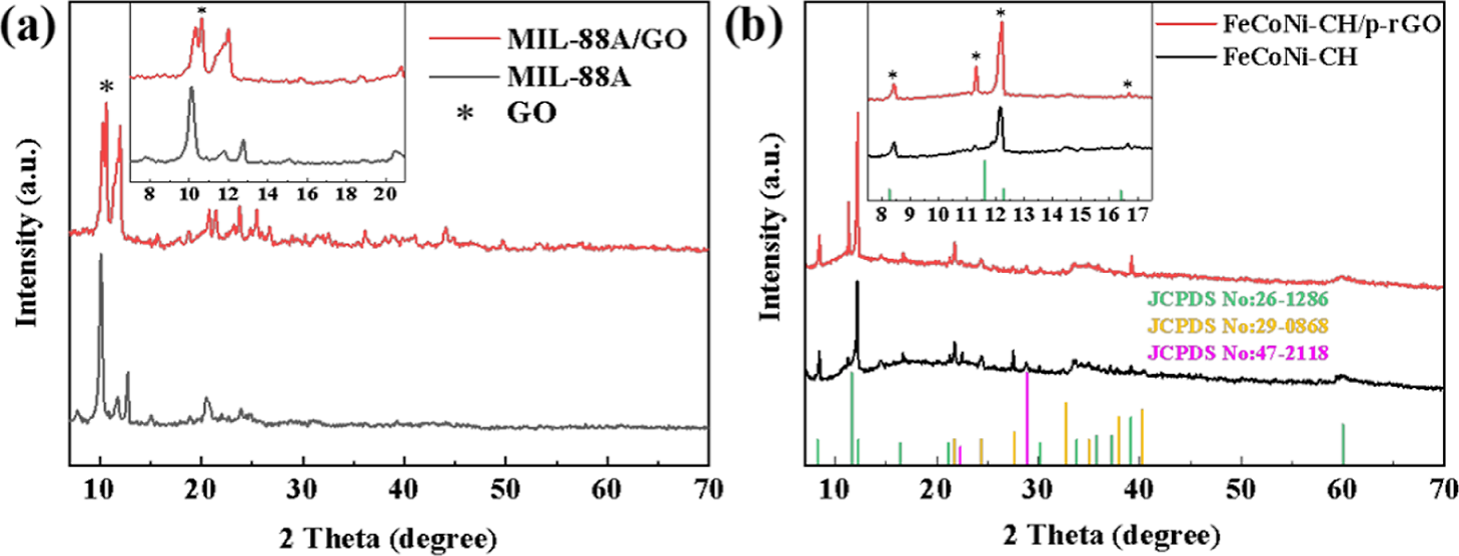
XRD diagrams of MIL-88A and MIL-88A/GO (a), FeCoNi–CH (10 h, the same below) and FeCoNi–CH/p-rGO (0.5 wt %, the same below) (b).

XRD is used to characterize the phases of the as-prepared materials. Four characteristic diffraction peaks at 2θ values of 7.7, 10.2, 12.8, and 15.1° are displayed in Figure 1a, which can be indexed to the (010), (011), (002), and (012) planes of the spindle-shaped MIL-88A phase.^34^ For the MIL-88A/GO, the diffraction peaks at 10.7° are indexed to the (001) of GO. Meanwhile, the disappearance of diffraction peaks at 7.7 and 12.8° and the appearance of some new diffraction peaks at 20–30° show that GO participates in coordination and forms new phases in the formation process of MIL-88A/GO.^35^ The diffraction peaks of FeCoNi–CH and FeCoNi–CH/p-rGO (Figure 1b) at 21.1, 30.2, 33.8, 35.7, 37.2, 39.1, and 60.0° are in good accordance with the Ni_6_Fe_2_(CO_3_)(OH)_16_·H_2_O phase (JCPDS no. 26-1286). Moreover, the peaks of 8.3, 11.6, 12.3, and 16.4° shift to a higher or lower angle than the simu_NiFe–CH, demonstrating that Co^2+^ has been successfully inset into the crystal lattice of FeNi–CH to cause a slight lattice expansion and shrinkage.^36^ Meanwhile, the diffraction peaks at 21.7, 24.4, 27.6, 32.7, 35.0, 37.9, and 40.2° can be indexed to the Ni_2_(CO_3_)(OH)_2_·H_2_O phase (JCPDS no. 29-0868), indicating successful preparation of FeCoNi–CH. In addition, the characteristic peaks at 22.3 and 28.9° come from residual fumaric acid (JCPDS no. 47-2118) during the reaction. The XRD patterns of materials synthesized in different conditions, including Co/Ni ratios, etching and coprecipitation times, and the amount of GO, are shown in Figures S1–S3.

**Figure 2:**
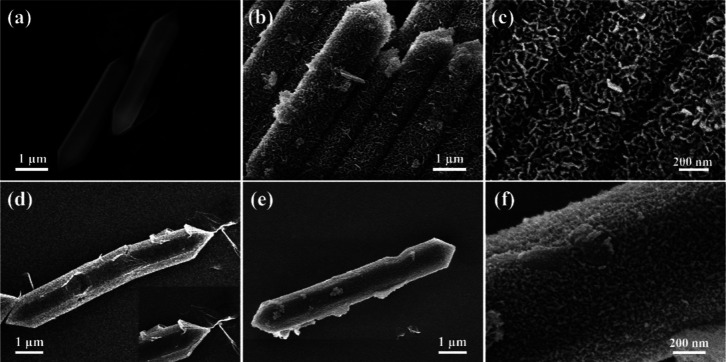
SEM images of MIL-88A (a) and FeCoNi–CH (b) and partial enlargement higher-resolution of FeCoNi–CH (c), MIL-88A/GO (d), and FeCoNi–CH/p-rGO (e) and partial enlargement higher-resolution of FeCoNi–CH/p-rGO (f).

The SEM images (Figure 2) reveal the morphology of the products. Both the precursor MIL-88A and MIL-88A/GO are fusiform (Figure 2a,d). By etching and coprecipitation, FeCoNi–CH retains the spindle shape of the MOF precursor, while the smooth surface of the precursor becomes a rough shell assembled from nanosheets (Figure 2b,c,e,f). In Figure 2d, some folded lamellar GO nanosheets are found, indicating that GO is composited. Furthermore, the EDS spectra (Figure S4) of FeCoNi–CH and FeCoNi–CH/p-rGO composite show that the contents of Co and Ni atoms are not consistent with the Co/Ni ratio of 1:1 added in the preparation process, and the concentration of Co is lower than that of Ni. There may be two reasons for the analysis: one is that the *K*_sp_ values of Co(OH)_2_ and Ni(OH)_2_ at the same temperature are different,^37^ resulting in different precipitation sequences, with Ni(OH)_2_ taking precedence over Co(OH)_2_. The other is that the nucleation rate of each ion in the reaction system is different during the formation of FeCoNi–CH, leading to a high content of metal elements with a fast nucleation rate in the FeCoNi–CH phase.

**Figure 3:**
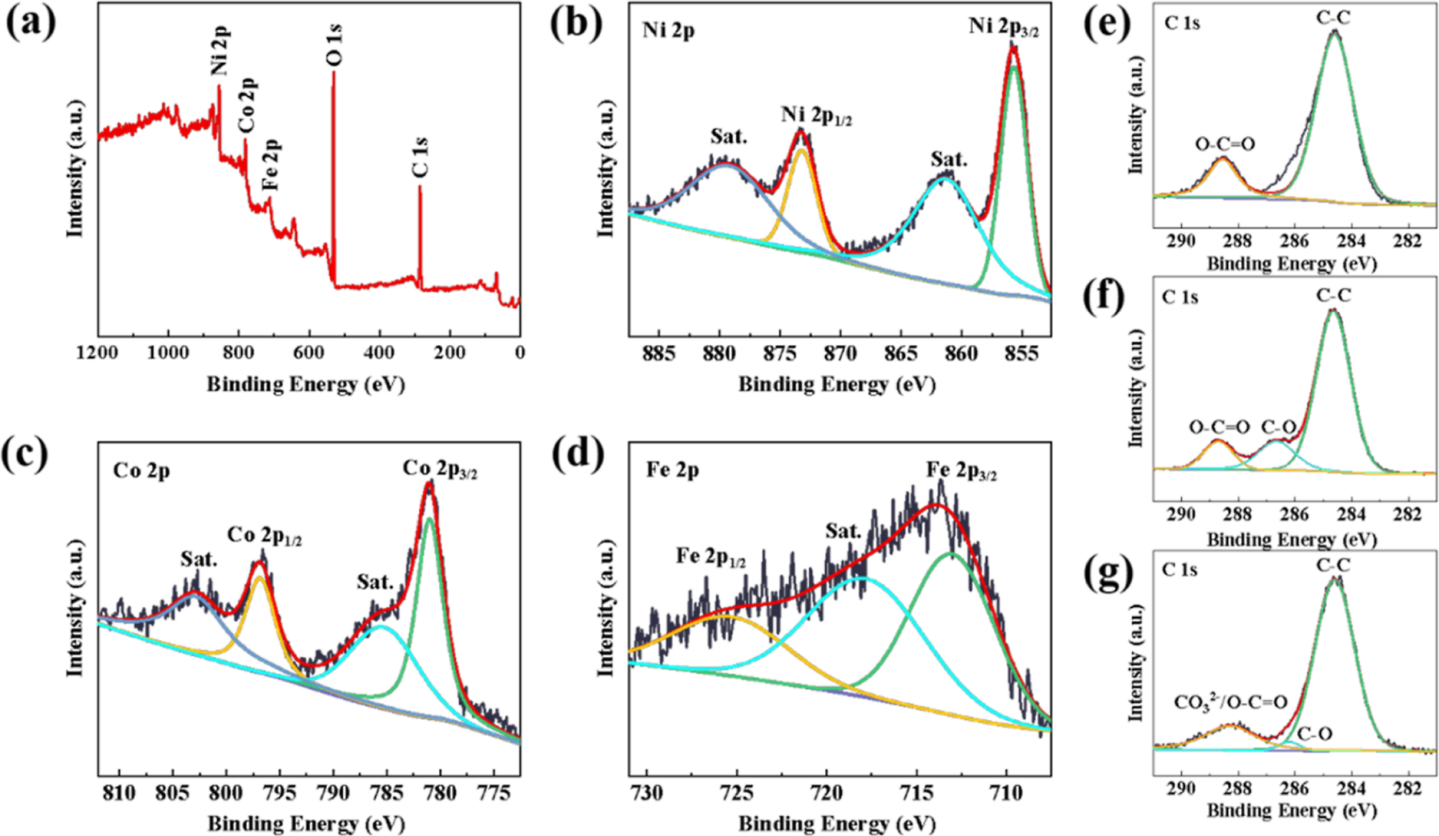
XPS spectra of the FeCoNi–CH/p-rGO composite: survey spectrum (a), Ni 2p (b), Co 2p (c), and Fe 2p (d); C 1s of MIL-88A (e), MIL-88A/GO (f), and FeCoNi–CH/p-rGO (g).

The chemical composition and valence state of the material surfaces of the MIL-88A, MIL-88A/GO, and FeCoNi–CH/p-rGO composites are characterized by XPS. In Figure 3a, the XPS survey spectrum of the FeCoNi–CH/p-rGO composite shows the Ni 2p, Co 2p, Fe 2p, O 1s, and C 1s peaks, which are very much in accord with the composition of FeCoNi–CH/p-rGO. As shown in Figure 3b, two main peaks representing Ni 2p_3/2_ and Ni 2p_1/2_ at 855.7 and 873.2 eV are separated by fitting. The gap in binding energy of 17.5 eV shows that the valence state of Ni is “+2”.^38^ In the same way, the spectrum of Co 2p (Figure 3c) can also be divided into two major peaks, standing for Co 2p_3/2_ and Co 2p_1/2_, respectively, at 780.98 and 796.83 eV and two satellite peaks, which indicates the valence state of Co is “+2”.^38^ Meanwhile, the two main peaks located at 713.0 and 725.3 eV in the Fe 2p spectrum (Figure 3d) demonstrate that the valence state of Fe is “+3”.^39^ As displayed in Figure 3e–g, the appearance of the oxygen-containing bond C–O in MIL-88A/GO proves the successful introduction of GO.^40^ The decrease in the intensity of C–O and the carboxyl peak of FeCoNi–CH/p-rGO indicates that GO is partially reduced to rGO by urea at 90 °C because the carboxyl and C–O groups do not disappear completely. This result may be caused by residual fumarate C_4_H_2_O_4_^2–^ and unreduced GO. Also, the offset of the binding energy position shows the presence of CO_3_^2–^ from FeCoNi–CH. The XPS spectra of MIL-88A and MIL-88A/GO are shown in Figure S5.

**Figure 4:**
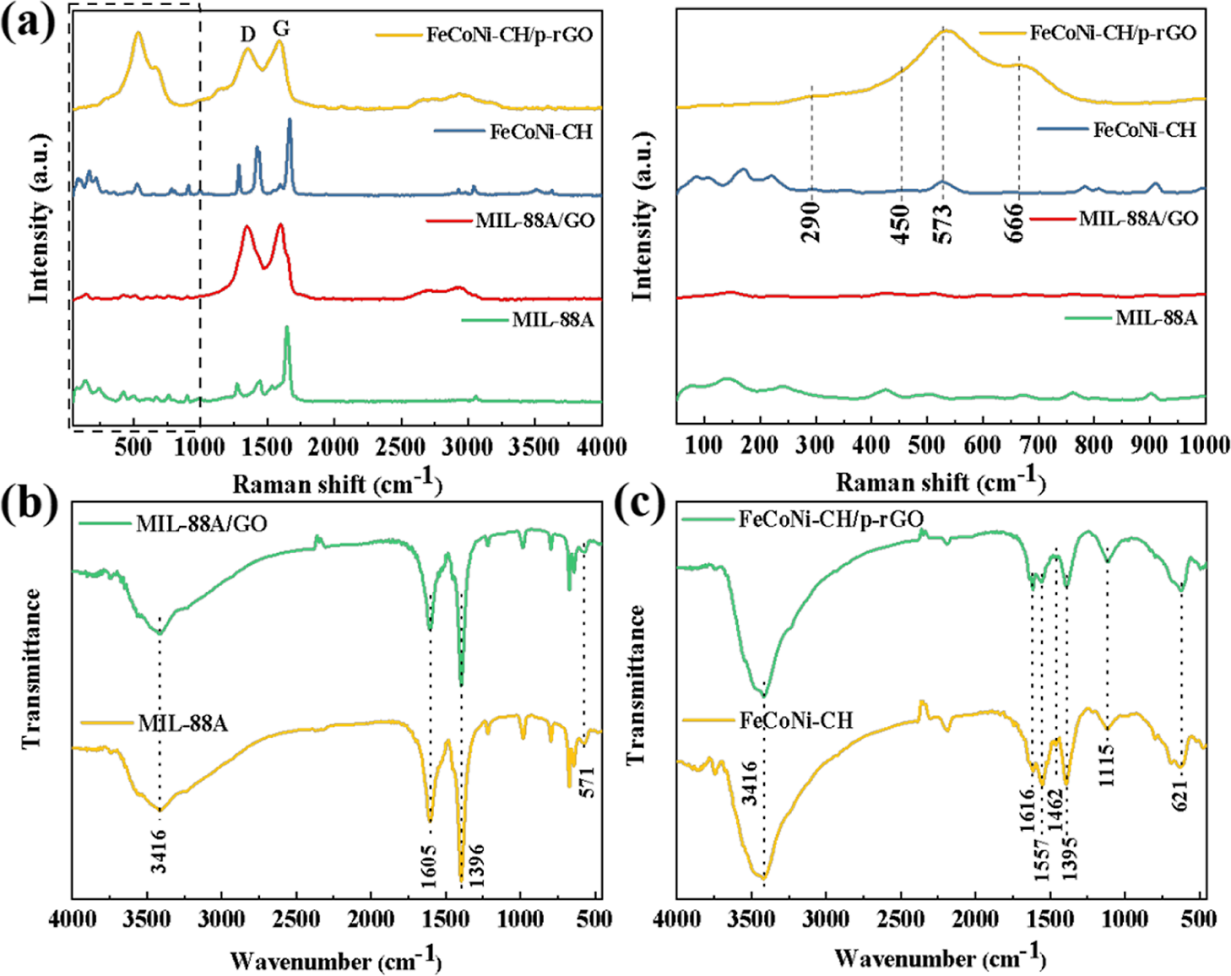
Raman spectra (a) and FT-IR spectra (b) of MIL-88A, MIL-88A/GO, and (c) FeCoNi–CH, FeCoNi–CH/p-rGO.

Raman spectroscopy is an effective characterization for analyzing the structural characteristics of carbonaceous materials, which can reflect the degree of reduction from GO to rGO. As shown in Figure 4a, the peak at 1661 cm^–1^ is caused by the stretching vibration of C=C in fumaric acid.^28^ In addition, the same high wavenumber peak also appears in the spectrum of FeCoNi–CH, which is attributed to the fumarate C_4_H_2_O_4_^2–^ isolated from MIL-88A during the etching process.^41^ This is exactly consistent with the XRD result, and it is necessary for C_4_H_2_O_4_^2–^ to balance the extra cationic charge in the layered CH.^42^ For MIL-88A/GO and FeCoNi–CH/p-rGO, two distinct main peaks are located at 1350 and 1598 cm^–1^, representing the D and G peaks of the carbonaceous material, respectively. The D peak is caused by the disorder and structural defect of sp^3^ carbon, while the G peak is caused by the plane vibration of ideal sp^2^ hybrid carbon. The relative strength ratio of peak D and peak G (*I*_D_/*I*_G_) is commonly used to measure the degree of graphitization and defect of the sample. The *I*_D_/*I*_G_ values of MIL-88A/GO and FeCoNi–CH/p-rGO were 1.39 and 1.90, respectively, indicating a larger degree of defects in rGO nanosheets despite the partial reduction of GO. On the one hand, when GO is reduced to rGO, the oxygen-containing functional groups of GO are removed, and the sp^2^ hybrid carbon-conjugated graphene can be rebuilt, which usually results in an increase in *I*_D_/*I*_G_ values.^43^ On the other hand, the increasing of the defect degree is also due to the interaction between rGO and FeCoNi–CH.^44^ Moreover, the peaks at 450, 537, 666, and 290 cm^–1^ in the FeCoNi–CH/p-rGO spectrum are attributed to Co–O, Ni–O, as well as Fe–O stretching vibration in CH and E-type, respectively. Moreover, the widening of the peak at 450 cm^–1^ arises from the defect and disordered structure of CH,^45^ which indicates the triumphant synthesis of the FeCoNi–CH/p-rGO composite.

The FT-IR spectra of MIL-88A, MIL-88A/GO, FeCoNi–CH, and FeCoNi–CH/p-rGO are exhibited in Figure 4b,c. The two similar wavenumbers of 1605 and 1396 cm^–1^ in MIL-88A and MIL-88A/GO represent the symmetric and asymmetric stretching vibrations of the carboxyl groups, respectively, and the wavenumber of 571 cm^–1^ represents the stretching vibration of Fe–O in MIL-88A. The wide peak at 3416 cm^–1^ wavenumber may be caused by residual water during the preparation of MIL-88A by hydrothermal method or water in the test environment,^34^ and the peak at 2350 cm^–1^ wavenumber is due to CO_2_ in the test environment.^46^ For FeCoNi–CH and FeCoNi–CH/p-rGO (Figure 4c), the wavenumbers at 1616, 1557, 1462, and 1395 cm^–1^ illustrate the existence of CO_3_^2–^. Therein, the bands at 1616 and 1557 cm^–1^ are attributed to the C=O and those at 1462 and 1395 cm^–1^ indicate the C–O. The CO_3_^2–^ is made up of one C=O and two C–O. Therefore, the wavenumber at 1115 cm^–1^ can be ascribed to the bending vibration of CO_3_^2–^. The wavenumber at 621 cm^–1^ is due to the overlapping of the asymmetric vibration of CO_3_^2–^ and to M–O stretching vibration.^47^

The type IV isotherms that come from N_2_ adsorption and desorption isotherms (Figure S6) depict mesoporous material, and the usual IV-H3 hysteresis indicates that the materials have a layered hollow structure, which is consistent with SEM images.^48^ The calculated Brunauer–Emmett–Teller (BET) specific surface areas of FeCoNi–CH and FeCoNi–CH/p-rGO composite are 30 and 27 m^2^ g^–1^, respectively, which demonstrates there are many active sites. The result of a slight decrease after compositing p-rGO is within the margin of test error and similar to the phenomenon reported in previous literature.^49^ The pore volumes of FeCoNi–CH and FeCoNi–CH/p-rGO composite calculated from Barrett–Joyner–Halenda (BJH) are 0.16 and 0.11 cm^3^ g^–1^ and the BJH pore diameters (Figure S6 inset) are within the range of 4 to 50 nm and 4 to 60 nm, respectively. The hierarchical porous structure allows more hydroxide ions to come into contact with the active substances and promotes electrochemical performance further.^50^

**Figure 5:**
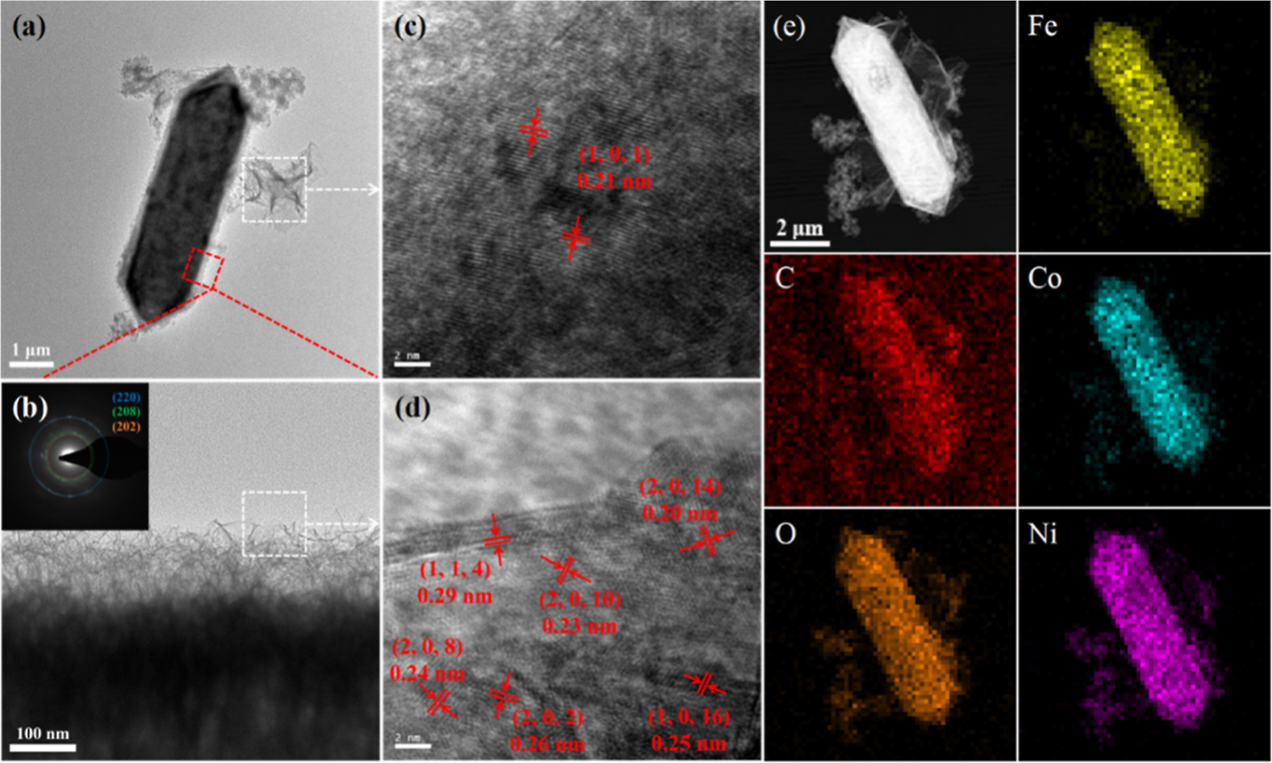
TEM images of FeCoNi–CH/p-rGO (a), partial enlargement of FeCoNi–CH/p-rGO (illustration is the selected area electron diffraction diagram) (b), HRTEM images of p-rGO (c) and FeCoNi–CH/p-rGO (d), and elemental mapping of FeCoNi–CH/p-rGO for C, O, Fe, Co, and Ni (e).

The TEM images of FeCoNi–CH/p-rGO are shown in Figure 5. It can be seen that the MOF template is etched, and FeCoNi–CH nanosheets are grown on the MOF substrates by self-assembly. Furthermore, the folding of the edges of the nanosheets demonstrates their ultrathin characteristics. As can be seen from Figure 5d, surface spacings of 0.20, 0.23, 0.24, 0.25, 0.26, and 0.29 nm are well corresponding to the (2, 0, 14), (2, 0, 10), (2, 0, 8), (1, 0, 16), (2, 0, 2), and (1, 1, 4) crystallographic planes of the FeCoNi–CH phase, respectively. The selection of electron diffraction in the illustration further proves the existence of the FeCoNi–CH phase. Meanwhile, the 0.21 nm crystal plane spacing corresponds to the GO (101) crystal plane in Figure 5c,^40^ indicating that GO has not been completely reduced. The mapping results (Figure 5e) exhibit that Fe, Co, and Ni elements are uniformly distributed, and the image of the C element indicates the existence of some fumarate C_4_H_2_O_4_^2–^ isolated from MOFs and CO_3_^2–^ in FeCoNi–CH layers.

### 3.3 Electrochemical Properties of the FeCoNi–CH and FeCoNi–CH/p-rGO Composite

The Co/Ni ratios and etching times are crucial to influence the electrochemical performance of FeCoNi–CH. Therefore, we regulate these factors to find that the optimal conditions for the electrochemical performance of the as-synthesized materials are a Co/Ni ratio of 1:1 and an etching time of 10 h (Figures S7 and S8). On this basis, the addition amount of GO is further regulated to 0.5 wt % to optimize the electrochemical performance of the FeCoNi–CH/p-rGO composites (Figure S9).

**Figure 6:**
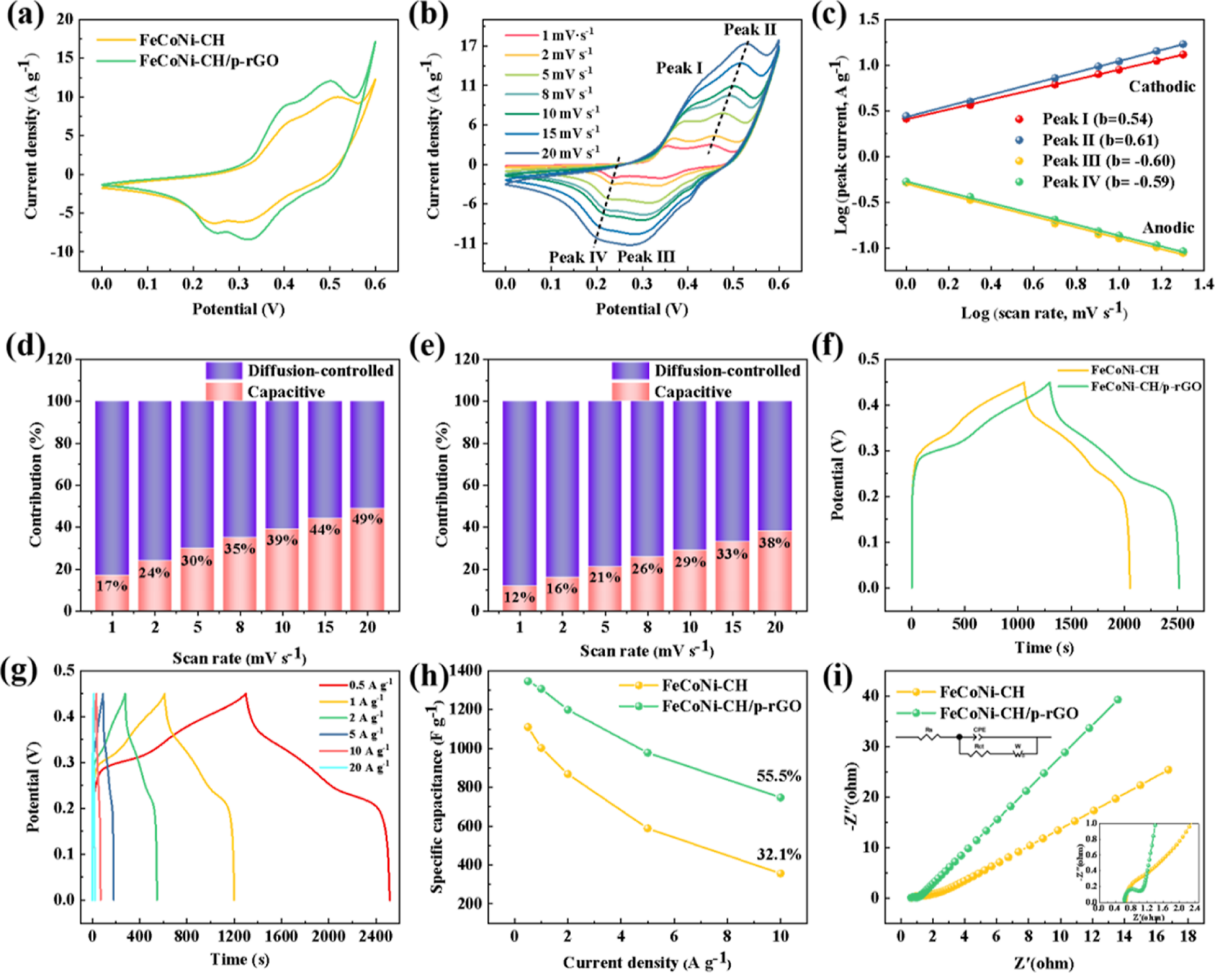
CV graphs of FeCoNi–CH and FeCoNi–CH/p-rGO at a sweep speed of 5 mV s^–1^ (a), CV graphs of FeCoNi–CH/p-rGO at different sweep speeds (b), image of linear fitting relationship between anode and cathode peak current and sweep speeds (c), relative contributions between these two charge storage mechanisms of capacitive and diffusion-controlled at different scan rates: FeCoNi–CH (d) and FeCoNi–CH/p-rGO (e), GCD graphs for FeCoNi–CH and FeCoNi–CH/p-rGO tested at a current density of 0.5 A g^–1^ (f), GCD graphs of FeCoNi–CH/p-rGO at various current densities (g), specific capacitance vs various current densities for FeCoNi–CH and FeCoNi–CH/p-rGO (h), and Nyquist curves of FeCoNi–CH and FeCoNi–CH/p-rGO with the inset of partial enlargement of high frequency region of the impedance spectrum (i).

To highlight the effect of GO on the electrochemical properties of composites, we performed the comparison between the FeCoNi–CH/p-rGO and FeCoNi–CH. As shown in Figure 6a, the CV curves of FeCoNi–CH and FeCoNi–CH/p-rGO at 5 mV s^–1^ have two similar pairs of distinct redox peaks, indicating that they are typical pseudocapacitive electrode materials. When the peak current increases (Figure 6b), the corresponding electrode potential also continuously shifts from the equilibrium potential and the polarization phenomenon occurs. The potential offset is linearly correlated with the peak current, so the polarization is mainly caused by the Ohm internal resistance in the electrode system.^51^ The CV curve (Figure 6a) also shows that the integral area of FeCoNi–CH/p-rGO is larger than that of FeCoNi–CH, manifesting that the specific capacitance of FeCoNi–CH/p-rGO is well improved.

Based on the analysis of CV curves, we further explore the charge storage mechanism of electrode materials with the reaction kinetic theory, which was first proposed by Simon et al.^56^ Reaction kinetics is used to express the relationship between peak current *i* and sweeping speed *v*
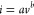
5where *a* and *b* are constants, and the value of *b* can be obtained by linear fitting of log(*i*) and log(*v*). In general, *b* = 0.5 demonstrates that a diffusion controlled battery-like mechanism is leading, and *b* = 1 demonstrates that capacitive behavior is dominant, here including electric double-layer capacitance and pseudocapacitance,^51^ and *b* = 0.5–1 manifests both the above processes.^52^ For FeCoNi–CH/p-rGO electrode materials, we label two pairs of redox peaks as peaks I, II, III, and IV (Figure 6b), and the corresponding *b* value is 0.54, 0.61, 0.60, and 0.59, respectively (Figure 6c). These results show that the diffusion-controlled battery-like behavior is dominant.^53^ The contribution proportion of both mechanisms can be quantified by the following two formulas
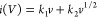
6
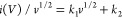
7where *i*(*V*) is the response current at different potentials (A g^–1^) and *k*_1_*v* and *k*_2_*v* correspond to capacitance process and diffusion control process, respectively. The values of *k*_1_ and *k*_2_ can be derived from the numerical relationship between *i*(*V*)/*v*^1/2^ and *v*^1/2^. For the composites FeCoNi–CH and FeCoNi–CH/p-rGO, the contribution ratios of the two mechanisms at varied scan rates are shown in Figure 6d,e, respectively. The similarity is that the proportion of the diffusion-controlled mechanism process decreases gradually with sweep speed increasing. Because the time of ion diffusion is shortened due to the rise in sweep speed, the number of ions inserted into the lattice is limited. On the contrary, the capacitance contribution will increase.^54^ The difference is that the capacitive contribution ratio of FeCoNi–CH/p-rGO is lower than that of FeCoNi–CH at the same scan rate. This may be due to the accelerated ion migration rate after rGO combination, which contributes to the occurrence of the diffusion-controlled mechanism process. Therefore, the proportion of the diffusion-controlled mechanism process is higher, and the capacitance contribution becomes low accordingly.

Based on the GCD curves shown in Figure 6f, the specific capacitance of the FeCoNi–CH and FeCoNi–CH/p-rGO composites are calculated to be 1110 and 1346 F g^–1^ at 0.5 A g^–1^, respectively. According to the GCD curves (Figure 6g), the specific capacitance of the FeCoNi–CH/p-rGO composites is calculated to be 1346, 1307, 1199, 978, 747, and 427 F g^–1^ at 0.5, 1, 2, 5, 10, and 20 A g^–1^, respectively. The capacitance retention rate (Figure 6h) of the FeCoNi–CH/p-rGO composites at 1–10 A g^–1^ is 55.5%, which is higher than that of FeCoNi–CH (32.1%). The reason for the high rate capability of FeCoNi–CH/p-rGO is that p-rGO can improve its electrical conductivity and facilitate rapid charge transfer.^55^

In order to study the speed of electron migration and ion diffusion in the Faraday redox process in an electrochemical system, EIS tests are implemented on the FeCoNi–CH and FeCoNi–CH/p-rGO composites. Figure 6i shows that the two curves consist of an arc in the high-frequency scope and an inclined straight line in the low-frequency scope, which is exactly similar to the AC impedance spectrum of the electrochemical system composed of pseudocapacitive electrode materials.^56^ In the high-frequency region, the intercept between the curve and the *X*-axis stands for *R*_s_, including the inherent resistance, electrolyte resistance, and interface contact resistance. The FeCoNi–CH/p-rGO exhibits the lower *R*_s_ value 0.63 Ω due to its higher conductivity than that of FeCoNi–CH (0.66 Ω). The semi-arc diameter of the high frequency scope stands for the electron transfer resistance *R*_ct_. According to the fitting result, the *R*_ct_ of FeCoNi–CH and FeCoNi–CH/p-rGO is 1.00 and 0.53 Ω, respectively, which means the electrical conductivity of the FeCoNi–CH/p-rGO is better. The low-frequency inclined straight line reflects the Warburg resistance of OH^–^ diffusing from the electrolyte to the surface of the electrode material. Moreover, the higher the slope is, the lower the ion diffusion resistance is. The fitting result is that the line slope of FeCoNi–CH/p-rGO is larger than that of FeCoNi–CH, which illustrates that the ion diffusion resistance is low and the ions are easy to diffuse and move in the electrolyte for FeCoNi–CH/p-rGO. The EIS analysis results show that FeCoNi–CH composed with p-rGO can improve the conductivity of the electrode material, and further promote the optimization of electrochemical properties. In addition, there is no obvious difference between the BET specific surface area and pore structure in FeCoNi–CH and FeCoNi–CH/p-rGO according to the above analysis, so the influence of improved conductivity after compositing p-rGO on the electrochemical properties is greater than that of the specific surface area and pore structure change.

**Figure 7:**
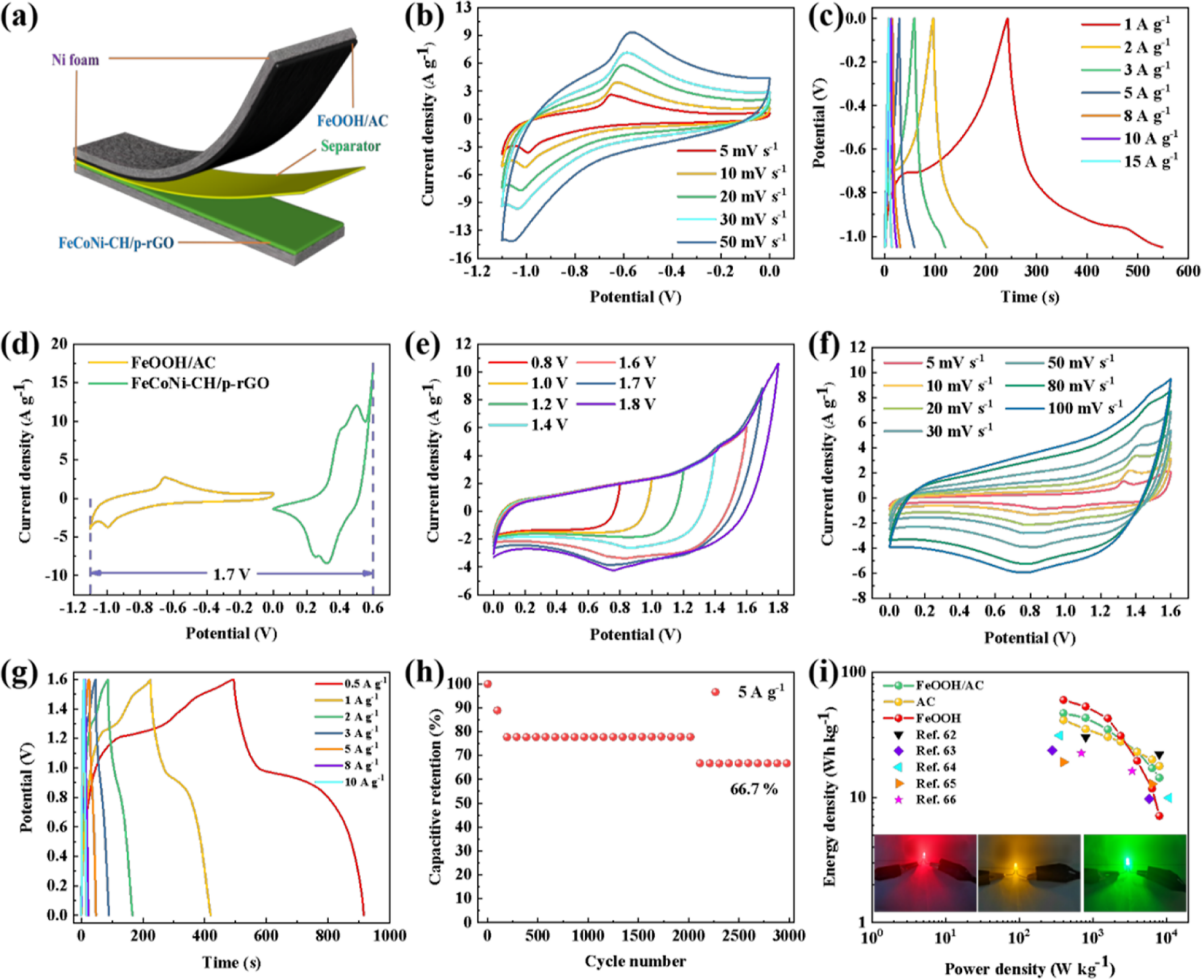
Sketch map of the FeCoNi–CH/p-rGO//FeOOH/AC HSC (a), CV graphs of FeOOH/AC at different sweep speeds in the scope of 5–50 mV s^–1^ (b), GCD graphs of FeOOH/AC at various current densities in the scope of 1–15 A g^–1^ (c), CV graphs of the FeCoNi–CH/p-rGO and FeOOH/AC electrodes measured at a sweep speed of 5 mV s^–1^ in a three-electrode system (d), CVs of as-prepared HSC tested at varied potential window with a sweep speed of 50 mV s^–1^ (e), electrochemical properties of the FeCoNi–CH/p-rGO//FeOOH/AC HSC: CV graphs at various sweep speeds (f), GCD graphs at different current densities (g), cycling performance (h), and Ragone plot between *E* and *P*, compared with other transition metal hydroxide-type HSCs from recent reports and the an illustration of lighting light-emitting diode (LED) through the as-prepared FeCoNi–CH/p-rGO//FeOOH/AC HSC (i).

High energy density and power density are the prerequisites for the practical application of supercapacitors.^57^ On the basis of previous studies, we constructed the composite FeCoNi–CH/p-rGO//FeOOH/AC HSC (Figure 7a). Compared to pure FeOOH or AC electrodes, assembling HSC with using FeCoNi–CH/p-rGO and FeOOH/AC as cathode and anode materials obtains better overall electrochemical performances.^58^ The CV (Figure 7b) and GCD (Figure 7c) curves under the voltage window of −1.1 to 0 V with varied scan rates reveal a pair of redox peaks and a potential platform in the low potential window, as well as a rectangular shape and an inclined straight shape, respectively, in the high potential window, which demonstrate that FeOOH/AC is a typical pseudocapacitance and electric double-layer capacitance binding electrode. At 1, 2, 3, 5, 8, 10, and 15 A g^–1^, the specific capacitance of FeOOH/AC is calculated to be 289, 201, 168, 136, 109, 91, and 68 F g^–1^, respectively. Calculated by eq 2, the optimized mass ratio of FeCoNi–CH/p-rGO to FeOOH/AC is 1:2. To determine the suitable voltage window for FeCoNi–CH/p-rGO//FeOOH/AC HSC, we perform the test for FeCoNi–CH/p-rGO and FeOOH/AC at 5 mV s^–1^. As displayed in Figure 7d, the wider voltage windows at 0–0.6 and −1.1 to 0 V are observed, which exceed the limiting voltage of hydrolysis (1.23 V).^59^ The CV curves (Figure 7e) of FeCoNi–CH/p-rGO//FeOOH/AC HSC at various voltage windows show the stable shape between 1.0 and 1.6 V. The slightly prominent redox peak in the curve indicates that the capacitance contribution of FeCoNi–CH/p-rGO//FeOOH/AC HSC is attributed to the pseudocapacitance of the cathode electrode and the electric double-layer capacitance as well as the pseudocapacitance of the anode electrode. When the voltage window rises above 1.7 V, polarization occurs, which illustrates that severe hydrolysis has occurred.^60^ Therefore, the optimal potential window is 1.6 V. With the rise of sweep speed, the CV curves still remain rectangular in shape (Figure 7f), and the polarization phenomenon is not obvious (Figure S10d,g). The GCD curves are triangular on the whole window with a few platforms (Figure 7g), which further proves that the electrochemical performance of the HSC comes from the pseudocapacitance characteristic of the cathode material and the electric double-layer as well as the pseudocapacitance characteristic of the anode material. At 0.5, 1, 2, 3, 5, 8, and 10 A g^–1^, the specific capacitance is calculated to be 132, 121, 98, 81, 65, 48, and 40 F g^–1^, respectively. Furthermore, the cycling stability of the FeCoNi–CH/p-rGO//FeOOH/AC HSC is detected at 5 A g^–1^ (Figure 7h). A high cycling stability of 66.7% is clearly exhibited, which falls between the 50% of FeCoNi–CH/p-rGO//FeOOH (shown in Figure S10i) and the 75% of FeCoNi–CH/p-rGO//AC (shown in Figure S10f) specific capacitance retention after cycling 3000 cycles. Step-like capacitance degradation in alkali environments may involve microstructure collapse caused by expansion and shrinkage, loss of available active sites, and resistance increase during charge–discharge processes.^61^ Therefore, the assembling of HSC using FeCoNi–CH/p-rGO and FeOOH/AC as cathode and anode materials can improve the overall electrochemical performance due to the synergy of pseudocapacitive material FeOOH and electric double-layer material AC.

According to eqs 3 and 4, the energy density and power density of the FeCoNi–CH/p-rGO//FeOOH/AC HSC device are computed, and the Ragone plot is shown in Figure 7i. The maximum energy density of 46.93 W h kg^–1^ of the device is delivered, which is between 59.73 W h kg^–1^ (FeCoNi–CH/p-rGO//FeOOH) and 41.33 W h kg^–1^ (FeCoNi–CH/p-rGO//AC), and the corresponding power density is 400 W kg^–1^. When the power density rises to 8000 W kg^–1^, the energy density can still be 14.30 W h kg^–1^. This result illustrates that the FeCoNi–CH/p-rGO//FeOOH/AC HSC device can maintain a high level of energy density under high current density and can achieve efficient, safe, and stable operation. Meanwhile, the FeCoNi–CH/p-rGO//FeOOH/AC HSC in this work precedes most of the other transition metal hydroxide-type HSCs reported previously, such as CoNiFe-LDH@CNFs//AC [*P* = 800 (8000) W kg^–1^, *E* = 30.2 (22.04) W h kg^–1^],^62^ NiCo-LDH on Zn_2_SnO_4_//AC [*P* = 283.4 (5817.2) W kg^–1^, *E* = 23.7 (9.7) W h kg^–1^],^63^ Ni–Co LDH/NG@AC [*P* = 354 (10 800) W kg^–1^, *E* = 31.2 (9.9) W h kg^–1^],^64^ CoMn-LDH/carbon fiber paper//AC [*P* = 400 (6400) W kg^–1^, *E* = 19.1 (12.8) W h kg^–1^],^65^ and rGO/NiMn-LDH/NF//AC [*P* = 700 (3425) W kg^–1^, *E* = 22.5 (16.2) W h kg^–1^].^66^Table 1 shows the comparisons of electrochemical performance intuitively.

**Table 1:** Comparison of Electrochemical Performance of the FeCoNi–CH/p-rGO Composites with Previous Reports

material	capacitance (F g^–1^)	current density (A g^–1^)	energy density (W h kg^–1^)	power density (W kg^–1^)	cycle performance	references
CoNiFe-LDH@CNFs	1203	1	30.2 (22.04)	800 (8000)	82.7%, 2000 cycle	(62)
NiCo-LDH/Zn_2_SnO_4_	1805	0.5	23.7 (9.7)	283 (5817)	92.7%, 5000 cycle	(63)
Ni–Co LDH/NG	1720	3	31.2 (9.9)	354 (10 800)	83%, 10 000 cycle	(64)
CoMn-LDH/CFP	980	2	19.1 (12.8)	400 (6400)		(65)
rGO/NiMn-LDH/NF	1696	1	22.5 (16.2)	700 (3425)	91%, 1000 cycle	(66)
FeCoNi–CH/p-rGO	1346	0.5	46.9 (14.3)	400 (8000)	66.7%, 3000 cycle	this work

## 4 Conclusions

In brief, FeCoNi–CH/p-rGO composites with a unique spindle form were effectively synthesized by a novel chemical approach employing urea as a precipitant and reducing agent, as well as MIL-88A as a self-template. The improved electrochemical performance of FeCoNi–CH/p-rGO composites is attributed to the pseudocapacitive energy storage capacity caused by the synergistic action of the ternary-metal CH and the high conductivity of p-rGO. Furthermore, the corresponding self-assembling FeCoNi–CH/p-rGO//FeOOH/AC HSC exhibits a high energy density of 46.93 W h kg^–1^ at 400 W kg^–1^ and cycle stability of 66.7% after 3000 cycles. This research provided a method for making MOF-derived ternary-metal CH/p-rGO electrode materials with various compositions and matrix morphologies, and the controllable synthesis materials could be able to expand their applications in the fields of electric energy storage, electrocatalysis, adsorption, and so forth. As a prospective research topic, the internal mechanisms impacting the electrochemical characteristics of the resultant materials under various preparation circumstances might be investigated further.

## Supporting Information Available

XRD patterns of FeCoNi–CH with different molar ratios of Co/Ni; XRD patterns of FeCoNi–CH with different etching times; XRD patterns of FeCoNi–CH/p-rGO with different ratios of GO; EDS spectra of FeCoNi–CH and FeCoNi–CH/p-rGO; XPS spectra of MIL-88A: survey spectra, Fe 2p, and O 1s, and MIL-88A/GO: survey spectra, Fe 2p, and O 1s; N_2_ adsorption and desorption isotherms and pore size distribution of the FeCoNi–CH and FeCoNi–CH/p-rGO; CV curves of FeCoNi–CH with different ratios of Co/Ni at a scan rate of 5 mV s^–1^; GCD curves for FeCoNi–CH with different ratios of Co/Ni tested at a current density of 0.5 A g^–1^; CV curves of FeCoNi–CH with Co/Ni ratio of 1:1 at different scan rates; GCD curves of FeCoNi–CH with Co/Ni ratio of 1:1 at different current densities; CV curves of FeCoNi–CH with different etching times at a scan rate of 5 mV s^–1^; GCD curves for FeCoNi–CH with different etching times at a current density of 0.5 A g^–1^; CV curves of FeCoNi–CH with etching time of 10 h at different scan rates; GCD curves of FeCoNi–CH with an etching time of 10 h at different current densities; CV curves of FeCoNi–CH/p-rGO with different ratios of GO at a scan rate of 5 mV s^–1^; GCD curves for FeCoNi–CH with different ratios of GO at a current density of 0.5 A g^–1^; CV curves of FeCoNi–CH/p-rGO with a GO content of 0.5 wt % at different scan rates; GCD curves of FeCoNi–CH/p-rGO with GO content of 0.5 wt % at different current densities; CV curves at a scan rate of 5 mV s^–1^, GCD curves at current density of 1 A g^–1^, and GCD curves at different current densities of AC, FeOOH, and FeOOH/AC; electrochemical performance of the FeCoNi–CH/p-rGO//AC HSC: CV curves at different scan rates, GCD curves at various current densities, and cycling performance at a current density of 5 A g^–1^; and electrochemical performance of the FeCoNi–CH/p-rGO//FeOOH HSC: CV curves at different scan rates, GCD curves at various current densities, and cycling performance at a current density of 5 A g^–1^ (PDF)

## References

[ref1] Li G.; Cai H.-R.; Li X.-L.; Zhang J.; Zhang D.-S.; Yang Y.-F.; Xiong J. Construction of Hierarchical NiCo_2_O_4_@Ni-MOF Hybrid Arrays on Carbon Cloth as Superior Battery-Type Electrodes for Flexible Solid-State Hybrid Supercapacitors. ACS Appl. Mater. Interfaces 2019, 11, 37675–37684. 10.1021/acsami.9b11994. 31532185

[ref2] Wang G.; Zhang L.; Zhang J. A Review of Electrode Materials for Electrochemical Supercapacitors. Chem. Soc. Rev. 2012, 41, 797–828. 10.1039/c1cs15060j. 21779609

[ref3] Wang X.; Yan C.; Sumboja A.; Yan J.; Lee P. S. Achieving High Rate Performance in Layered Hydroxide Supercapacitor Electrodes. Adv. Energy Mater. 2014, 4, 1301240 10.1002/aenm.201301240.

[ref4] Wang L.; Liu F.; Pal A.; Ning Y.; Wang Z.; Zhao B.; Bradley R.; Wu W. Ultra-Small Fe_3_O_4_ Nanoparticles Encapsulated in Hollow Porous Carbon Nanocapsules for High Performance Supercapacitors. Carbon 2021, 179, 327–336. 10.1016/j.carbon.2021.04.024.

[ref5] Wang Z.; Liu Y.; Gao C.; Jiang H.; Zhang J. A Porous Co(OH)_2_ Material Derived from MOFs Template and Its Superior Energy Storage Performances for Supercapacitors. J. Mater. Chem. A 2015, 3, 20658–20663. 10.1039/c5ta04663g.

[ref6] Le K.; Gao M.; Liu W.; Liu J.; Wang Z.; Wang F.; Murugadoss V.; Wu S.; Ding T.; Guo Z. MOF-Derived Hierarchical Core-Shell Hollow Iron-Cobalt Sulfides Nanoarrays on Ni Foam with Enhanced Electrochemical Properties for High Energy Density Asymmetric Supercapacitors. Electrochim. Acta 2019, 323, 134826 10.1016/j.electacta.2019.134826.

[ref7] El Sharkawy H. M.; Sayed D. M.; Dhmees A. S.; Aboushahba R. M.; Allam N. K. Facile Synthesis of Nanostructured Binary Ni-Cu Phosphides as Advanced Battery Materials for Asymmetric Electrochemical Supercapacitors. ACS Appl. Energy Mater. 2020, 3, 9305–9314. 10.1021/acsaem.0c01630.

[ref8] Sun P.-P.; Zhang Y.-H.; Shi H.; Shi F.-N. Controllable One Step Electrochemical Synthesis of PANI Encapsulating 3d-4f Bimetal MOFs Heterostructures as Electrode Materials for High-Performance Supercapacitors. Chem. Eng. J. 2022, 427, 130836 10.1016/j.cej.2021.130836.

[ref9] Jiang D.; Wei C.-Y.; Zhu Z.-Y.; Guan X.-H.; Lu M.; Zhang X.-J.; Wang G.-S. Synthesis of 3D Flower-Like Hierarchical NiCo-LDH Microspheres with Boosted Electrochemical Performance for Hybrid Supercapacitors. Inorg. Chem. Front. 2021, 8, 4324–4333. 10.1039/d1qi00613d.

[ref10] Zhang X.; Yi H.; An Q.; Song S. Recent Advances in Engineering Cobalt Carbonate Hydroxide for Enhanced Alkaline Water Splitting. J. Alloys Compd. 2021, 887, 161405 10.1016/j.jallcom.2021.161405.

[ref11] Xiong S.; Chen J.-S.; Lou X.-W.; Zeng H.-C. Mesoporous Co_3_O_4_ and CoO@C Topotactically Transformed from Chrysanthemum-Like Co(CO_3_)_0.5_(OH)·0.11H_2_O and Their Lithium-Storage Properties. Adv. Funct. Mater. 2012, 22, 861–871. 10.1002/adfm.201102192.

[ref12] Joshi S.; Jones L. A.; Sabri Y. M.; Bhargava S. K.; Sunkara M. V.; Ippolito S. J. Facile Conversion of Zinc Hydroxide Carbonate to CaO-ZnO for Selective CO_2_ Gas Detection. J. Colloid Interface Sci. 2020, 558, 310–322. 10.1016/j.jcis.2019.09.103. 31605933

[ref13] Li X.; Tang Y.; Zhu J.; Lv H.; Zhao L.; Wang W.; Zhi C.; Li H. Boosting the Cycling Stability of Aqueous Flexible Zn Batteries Via F Doping in Nickel–Cobalt Carbonate Hydroxide Cathode. Small 2020, 16, 2001935 10.1002/smll.202001935. 32603014

[ref14] Zheng X.; Ye Y.; Yang Q.; Geng B.; Zhang X. Ultrafine Nickel–Copper Carbonate Hydroxide Hierarchical Nanowire Networks for High-Performance Supercapacitor Electrodes. Chem. Eng. J. 2016, 290, 353–360. 10.1016/j.cej.2016.01.076.

[ref15] Cai J.; Huang J.; Xu S.; Yuan L.; Huang X.; Huang Z.; Zhang C. Nickel Iron Carbonate Hydroxide Hydrate Decorated with CeO_x_ for Highly Efficient Oxygen Evolution Reaction. J. Solid State Electrochem. 2019, 23, 3449–3458. 10.1007/s10008-019-04445-9.

[ref16] Li Y.; Chen J.; Cai P.; Wen Z. An Electrochemically Neutralized Energy-Assisted Low-Cost Acid-Alkaline Electrolyzer for Energy-Saving Electrolysis Hydrogen Generation. J. Mater. Chem. A 2018, 6, 4948–4954. 10.1039/c7ta10374c.

[ref17] Zhong Y.; Cao X.; Liu Y.; Cui L.; Liu J. Nickel Cobalt Manganese Ternary Carbonate Hydroxide Nanoflakes Branched on Cobalt Carbonate Hydroxide Nanowire Arrays as Novel Electrode Material for Supercapacitors with Outstanding Performance. J. Colloid Interface Sci. 2021, 581, 11–20. 10.1016/j.jcis.2020.07.124. 32771723

[ref18] Zhang Q.; Liu Z.; Zhao B.; Cheng Y.; Zhang L.; Wu H.-H.; Wang M.-S.; Dai S.; Zhang K.; Ding D.; Wu Y.; Liu M. Design and Understanding of Dendritic Mixed-Metal Hydroxide Nanosheets@N-doped Carbon Nanotube Array Electrode for High-Performance Asymmetric Supercapacitors. Energy Storage Mater. 2019, 16, 632–645. 10.1016/j.ensm.2018.06.026.

[ref19] Wang J.; Yang L.; Fu Y.; Yin P.; Guan X.; Wang G. Delicate Control of Crystallographic Cu_2_O Derived Ni-Co Amorphous Double Hydroxide Nanocages for High-Performance Hybrid Supercapacitors: an Experimental and Computational Investigation. Nanoscale 2021, 13, 8562–8574. 10.1039/d1nr01016f. 33912892

[ref20] Liu H.; Liu N.; Lu M. Research Progress of Metal-Organic Frameworks in the Adsorption Removal of Heavy Metal Ions from Wastewater. J. Northeast Electr. Power Univ. 2019, 39, 58–66.

[ref21] Zhao J.; Chen J.; Xu S. Hierarchical NiMn Layered Double Hydroxide/Carbon Nanotubes Architecture with Superb Energy Density for Flexible Supercapacitors. Adv. Funct. Mater. 2014, 24, 2938–2946. 10.1002/adfm.201303638.

[ref22] Warsi M. F.; Shakir I.; Shahid M.; Sarfraz M.; Nadeem M.; Gilani Z. A. Conformal Coating of Cobalt-Nickel Layered Double Hydroxides Nanoflakes on Carbon Fibers for High-Performance Electrochemical Energy Storage Supercapacitor Devices. Electrochim. Acta 2014, 135, 513–518. 10.1016/j.electacta.2014.05.020.

[ref23] Yang T.; Ye Q.; Liang Y.; Wu L.; Long X.; Xu X.; Wang F. Graded Holey Nickel Cobalt Layered Double Hydroxide Nanosheet Array Electrode with High Mass Loading for High-Energy-Density All-Solid-State Supercapacitors. J. Power Sources 2020, 449, 227590 10.1016/j.jpowsour.2019.227590.

[ref24] Wang Z.; Zhang X.; Wang J.; Zou L.; Liu Z.; Hao Z. Preparation and Capacitance Properties of Graphene/NiAl Layered Double-Hydroxide Nanocomposite. J. Colloid Interface Sci. 2013, 396, 251–257. 10.1016/j.jcis.2013.01.013. 23411355

[ref25] Lu M.; Sun M.; Guan X.; Chen X.; Wang G. S. Controllable Synthesis of Hollow Spherical Nickel Chalcogenide (NiS_2_ and NiSe_2_) Decorated with Graphene for Efficient Supercapacitor Electrodes. RSC Adv. 2021, 11, 11786–11792. 10.1039/d0ra10659c. 35423764 PMC8696559

[ref26] Shin H.; Kim K. K.; Benayad A.; Yoon S.; Park H. K.; Jung I.; Jin M. H.; Jeong H.; Kim J. M.; Choi J.; Lee Y. H. Efficient Reduction of Graphite Oxide by Sodium Borohydride and Its Effect on Electrical Conductance. Adv. Funct. Mater. 2009, 19, 1987–1992. 10.1002/adfm.200900167.

[ref27] Moon I. K.; Lee J.; Ruoff R. S. Reduced Graphene Oxide by Chemical Graphitization. Nat. Commun. 2010, 1, 73 10.1038/ncomms1067. 20865806

[ref28] Wang J.; Wan J.; Ma Y.; Wang Y.; Pu M.; Guan Z. Metal–Organic Frameworks MIL-88A with Suitable Synthesis Conditions and Optimal Dosage for Effective Catalytic Degradation of Orange G through Persulfate Activation. RSC Adv. 2016, 6, 112502–112511. 10.1039/c6ra24429g.

[ref29] Pei C.; Liu C.; Wang Y.; Cheng D.; Li R.; Shu W.; Zhang C.; Hu W.; Jin A.; Yang Y. FeOOH@Metal-Organic Framework Core-Satellite Nanocomposites for the Serum Metabolic Fingerprinting of Gynecological Cancers. Angew. Chem., Int. Ed. 2020, 59, 10831–10835. 10.1002/anie.202001135. 32237260

[ref30] Chakrabarty S.; Mukherjee A.; Su W. N.; Basu S. Improved Bi-functional ORR and OER Catalytic Activity of Reduced Graphene Oxide Supported ZnCo_2_O_4_ Microsphere. Int. J. Hydrogen Energy 2019, 44, 1565–1578. 10.1016/j.ijhydene.2018.11.163.

[ref31] Fan X. B.; Peng W. C.; Li Y. Deoxygenation of Exfoliated Graphite Oxide under Alkaline Conditions: A Green Route to Graphene Preparation. Adv. Mater. 2008, 20, 4490–4493. 10.1002/adma.200801306.

[ref32] Lee J. H.; Shin W. H.; Ryou M. H. Functionalized Graphene for High Performance Lithium Ion Capacitors. ChemSusChem 2012, 5, 2328–2333. 10.1002/cssc.201200549. 23112143

[ref33] Lei Z.; Lu L.; Zhao X. S. The Electrocapacitive Properties of Graphene Oxide Reduced by Urea. Energy Environ. Sci. 2012, 5, 6391 10.1039/c1ee02478g.

[ref34] Zhang Y.; Zhou J.; Chen X.; Wang L.; Cai W. Coupling of Heterogeneous Advanced Oxidation Processes and Photocatalysis in Efficient Degradation of Tetracycline Hydrochloride by Fe-Based MOFs: Synergistic Effect and Degradation Pathway. Chem. Eng. J. 2019, 369, 745–757. 10.1016/j.cej.2019.03.108.

[ref35] Liu X.; Zhou H.; Zhang Y.; Liu Y.; Yuan A. Syntheses, Characterizations and Adsorption Properties of MIL-101/Graphene Oxide Composites. Chin. J. Chem. 2012, 30, 2563–2566. 10.1002/cjoc.201200575.

[ref36] Ding J. Y.; Zhong L.; Huang Q. Chitosan Hydrogel Derived Carbon Foam with Typical Transition-metal Catalysts for Efficient Water Splitting. Carbon 2021, 177, 160–170. 10.1016/j.carbon.2021.01.160.

[ref37] Shang Y.; Ma S.; Wei Y.; Yang H.; Xu Z. Flower-Like Ternary Metal of Ni-Co-Mn Hydroxide Combined with Carbon Nanotube for Supercapacitor. Ionics 2020, 26, 3609–3619. 10.1007/s11581-020-03496-7.

[ref38] Dong C.; Han L.; Zhang C.; Zhang Z. Scalable Dealloying Route to Mesoporous Ternary CoNiFe Layered Double Hydroxides for Efficient Oxygen Evolution. ACS Sustainable Chem. Eng. 2018, 6, 16096–16104. 10.1021/acssuschemeng.8b02656.

[ref39] Zhang J.; Yu L.; Chen Y.; Lu X.-F.; Gao S.; Lou X.-W. Designed Formation of Double-Shelled Ni-Fe Layered-Double-Hydroxide Nanocages for Efficient Oxygen Evolution Reaction. Adv. Mater. 2020, 32, 1906432 10.1002/adma.201906432. 32134141

[ref40] Lellala K. Microwave-Assisted Facile Hydrothermal Synthesis of Fe_3_O_4_-GO Nanocomposites for the Efficient Bifunctional Electrocatalytic Activity of OER/ORR. Energy Fuels 2021, 35, 8263–8274. 10.1021/acs.energyfuels.0c04411.

[ref41] Sun H.; Chen L.; Lian Y.; Yang W.; Lin L.; Chen Y.; Xu J.; Wang D.; Yang X.; Rümmerli M. H.; Guo J.; Zhong J.; Deng Z.; Jiao Y.; Peng Y.; Qiao S. Topotactically Transformed Polygonal Mesopores on Ternary Layered Double Hydroxides Exposing Under-Coordinated Metal Centers for Accelerated water Dissociation. Adv. Mater. 2020, 32, 2006784 10.1002/adma.202006784. 33184955

[ref42] Wang Q.; O’Hare D. Recent Advances in the Synthesis and Application of Layered Double Hydroxide (LDH) Nanosheets. Chem. Rev. 2012, 112, 4124–4155. 10.1021/cr200434v. 22452296

[ref43] Cai X.; Shen X.; Ma L.; Ji Z.; Xu C.; Yuan A. Solvothermal Synthesis of NiCo-Layered Double Hydroxide Nanosheets Decorated on RGO Sheets for High Performance Supercapacitor. Chem. Eng. J. 2015, 268, 251–259. 10.1016/j.cej.2015.01.072.

[ref44] Shi Z. T.; Sun G. Y.; Rui W. Scalable Fabrication of NiCo2O4/reduced Graphene Oxide Composites by Ultrasonic Spray as Binder-free Electrodes for Supercapacitors with Ultralong Lifetime. J. Mater. Sci. Technol. 2022, 99, 260–269. 10.1016/j.jmst.2021.05.040.

[ref45] Louie M. W.; Bell A. T. An Investigation of Thin-Film Ni-Fe Oxide Catalysts for the Electrochemical Evolution of Oxygen. J. Am. Chem. Soc. 2013, 135, 12329–12337. 10.1021/ja405351s. 23859025

[ref46] Yang P.; Wu Z.; Jiang Y.; Pan Z.; Tian W.; Jiang L.; Hu L. Fractal (Ni_x_Co_1-x_)_9_Se_8_ Nanodendrite Arrays with Highly Exposed (01-1) Surface for Wearable, All-Solid-State Supercapacitor. Adv. Energy Mater. 2018, 8, 1801392 10.1002/aenm.201801392.

[ref47] Lee Y.; Choi J. H.; Jeon H. J.; Choi K. M.; Lee J. W.; Kang J. K. Titanium-Embedded Layered Double Hydroxides as Highly Efficient Water Oxidation Photocatalysts Under Visible Light. Energy Environ. Sci. 2011, 4, 914–920. 10.1039/c0ee00285b.

[ref48] Thommes M.; Kaneko K.; Neimark A. V. Physisorption of Gases, with Special Reference to the Evaluation of Surface Area and Pore Size Distribution (IUPAC Technical Report). Pure Appl. Chem. 2015, 87, 1051–1069. 10.1515/pac-2014-1117.

[ref49] Petit C.; Bandosz T. J. MOF-Graphite Oxide Nanocomposites: Surface Characterization and Evaluation as Adsorbents of Ammonia. J. Mater. Chem. 2009, 19, 6521–6528. 10.1039/b908862h.

[ref50] Zhu Z. Y.; Wei C. Y.; Jiang D. Design and Synthesis of MOF-derived CuO/g-C_3_N_4_ Composites with Octahedral Structures as Advanced Anode Materials for Asymmetric Supercapacitors with High Energy and Power Densities. Mater. Adv. 2022, 3, 672 10.1039/d1ma00766a.

[ref51] Pu X.; Zhao D.; Fu C.; Chen Z.; Cao S.; Wang C.; Cao Y. Understanding and Calibration of Charge Storage Mechanism in Cyclic Voltammetry Curves. Angew. Chem., Int. Ed. 2021, 60, 21310–21318. 10.1002/anie.202104167. 34254416

[ref52] Liu J.; Wang J.; Xu C.; Jiang H.; Li C.; Zhang L.; Lin J.; Shen Z. X. Advanced Energy Storage Devices: Basic Principles, Analytical Methods, and Rational Materials Design. Adv. Sci. 2018, 5, 1700322 10.1002/advs.201700322. PMC5770679 29375964

[ref53] Lu W.; Shen J.; Zhang P.; Zhong Y.; Hu Y.; Lou X.-W. Construction of CoO/Co-Cu-S Hierarchical Tubular Heterostructures for Hybrid Supercapacitors. Angew. Chem., Int. Ed. 2019, 58, 15441–15447. 10.1002/anie.201907516. 31380596

[ref54] Wang R.; Xu C.; Lee J. M. High Performance Asymmetric Supercapacitors: New NiOOH Nanosheet/Graphene Hydrogels and Pure Graphene Hydrogels. Nano Energy 2016, 19, 210–221. 10.1016/j.nanoen.2015.10.030.

[ref55] Wu Y.; Yan D.; Zhang Z. Electron Highways into Nanochannels of Covalent Organic Frameworks for High Electrical Conductivity and Energy Storage. ACS Appl. Mater. Interfaces 2019, 11, 7661–7665. 10.1021/acsami.8b21696. 30702269

[ref56] Augustyn V.; Simon P.; Dunn B. Pseudocapacitive Oxide Materials for High-Rate Electrochemical Energy Storage. Energy Environ. Sci. 2014, 7, 1597–1614. 10.1039/c3ee44164d.

[ref57] Wang J.; Wang Z.; Tian X. Research on Braking Energy Storage System Based on Super-Capacitor for Pumping Unit. J. Northeast Electr. Power Univ. 2021, 41, 99–106.

[ref58] Sun G.; Ren H.; Shi Z. T. V_2_O_5_/Vertically-aligned Carbon Nanotubes as Negative Electrode for Asymmetric Supercapacitor in Neutral Aqueous Electrolyte. J. Colloid Interface Sci. 2021, 588, 847–856. 10.1016/j.jcis.2020.11.126. 33309246

[ref59] Wang T.; Zhang S.; Yan X. 2-Methylimidazole-Derived Ni-Co Layered Double Hydroxide Nanosheets as High Rate Capability and High Energy Density Storage Material in Hybrid Supercapacitors. ACS Appl. Mater. Interfaces 2017, 9, 15510–15524. 10.1021/acsami.7b02987. 28430411

[ref60] Saraf M.; Rajak R.; Mobin S. M. A Fascinating Multitasking Cu-MOF/rGO Hybrid for High Performance Supercapacitors and Highly Sensitive and Selective Electrochemical Nitrite Sensors. J. Mater. Chem. A 2016, 4, 16432–16445. 10.1039/c6ta06470a.

[ref61] Wang G. L.; Huang J. C.; Chen S. L.; Gao Y. Y.; Cao D. X. Preparation and Supercapacitance of CuO Nanosheet Arrays Grown on Nickel Foam. J. Power Sources 2011, 196, 5756–5760. 10.1016/j.jpowsour.2011.02.049.

[ref62] Wang F.; Sun S.; Xu Y.; Wang T.; Yu R.; Li H. High Performance Asymmetric Supercapacitor Based on Cobalt Nickle Iron-Layered Double Hydroxide/Carbon Nanofibres and Activated Carbon. Sci. Rep. 2017, 7, 4707 10.1038/s41598-017-04807-1. 28680040 PMC5498571

[ref63] Wang X.; Sumboja A.; Lin M.; Yan J.; Lee P. S. Enhancing Electrochemical Reaction Sites in Nickel-Cobalt Layered Double Hydroxides on Zinc Tin Oxide Nanowires: a Hybrid Material for an Asymmetric Supercapacitor Device. Nanoscale 2012, 4, 7266–7272. 10.1039/c2nr31590d. 23076678

[ref64] Wang W.; Zhang N.; Ye Z.; Hong Z.; Zhi M. Synthesis of 3D Hierarchical Porous Ni-Co Layered Double Hydroxide/N-doped Reduced Graphene Oxide Composites for Supercapacitor Electrodes. Inorg. Chem. Front. 2019, 6, 407–416. 10.1039/c8qi01132j.

[ref65] Zhao C.; Tian S.; Nie P.; Deng T.; Ren F.; Chang L. Electrodeposited Binder-Free CoMn LDH/CFP Electrode with High Electrochemical Performance for Asymmetric Supercapacitor. Ionics 2020, 26, 1389–1396. 10.1007/s11581-019-03290-0.

[ref66] Sun L.; Zhang Y.; Zhang Y.; Si H.; Qin W.; Zhang Y. Reduced Graphene Oxide Nanosheet Modified NiMn-LDH Nanoflake Arrays for High-Performance Supercapacitors. Chem. Commun. 2018, 54, 10172–10175. 10.1039/c8cc05745a. 30137073

