# Supplementary material for: Skillful Introduction of Urea during the Synthesis of MOF-Derived FeCoNi–CH/p-rGO with a Spindle-Shaped Substrate for Hybrid Supercapacitors
Source: ACS Omega. 2022 Sep 6;7(37):33019–30. doi: 10.1021/acsomega.2c02712 (PMC9494635; doi:10.1021/acsomega.2c02712)
Supplement: Supplementary file 1 — ao2c02712_si_001.pdf [file ao2c02712_si_001.pdf]

Supporting Information

Skillful introduction of urea during the synthesis of MOFs-derived FeCoNi-CH/p-rGO with a spindle-shaped substrate for hybrid supercapacitors

Yu Zhang, [a] *Chen-Ming Liang,* [a] *Min Lu,* *[a] *Hao Yu,* *[a] *and Guang-Sheng Wang* *[b]

a. School of Chemical Engineering, Northeast Electric Power University, Jilin 132000, China.

b. School of Chemistry, Beihang University, Beijing 100191, China.

Corresponding Author

Min Lu, E-mail: 19770919@163.com

Hao Yu, E-mail: yuh123@126.com

Guang-Sheng Wang, E-mail: wanggsh@buaa.edu.cn

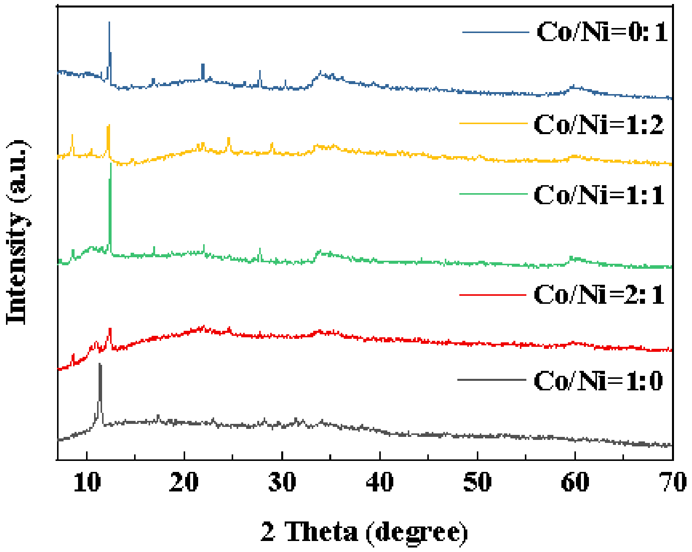

Figure S1. XRD patterns of FeCoNi-CH with different molar ratios of Co/Ni.

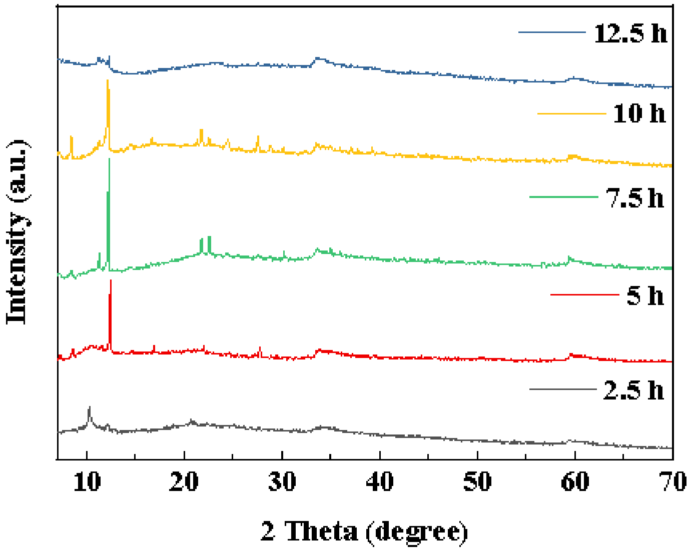

Figure S2. XRD patterns of FeCoNi-CH with different etching times.

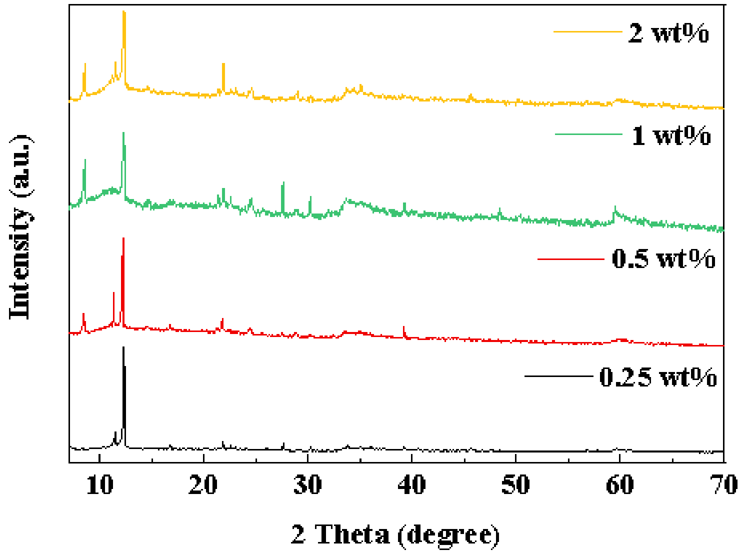

Figure S3. XRD patterns of FeCoNi-CH/p-rGO with different ratios of GO.

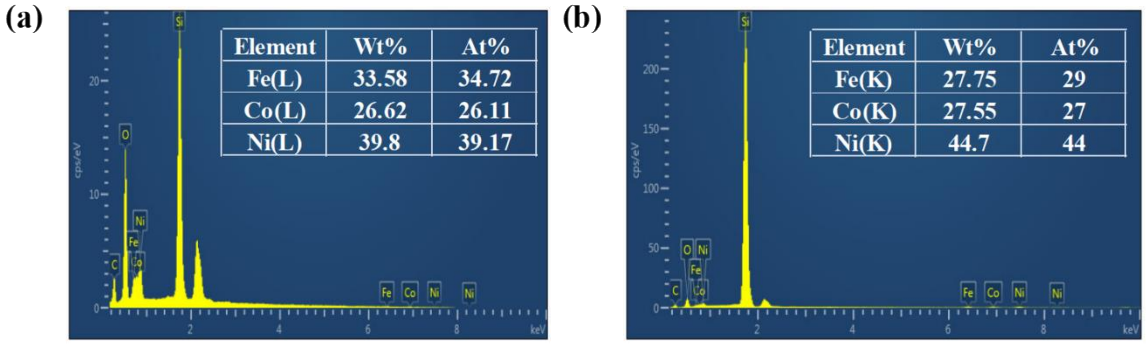

Figure S4. EDS spectra of FeCoNi-CH (a) and FeCoNi-CH/p-rGO (b).

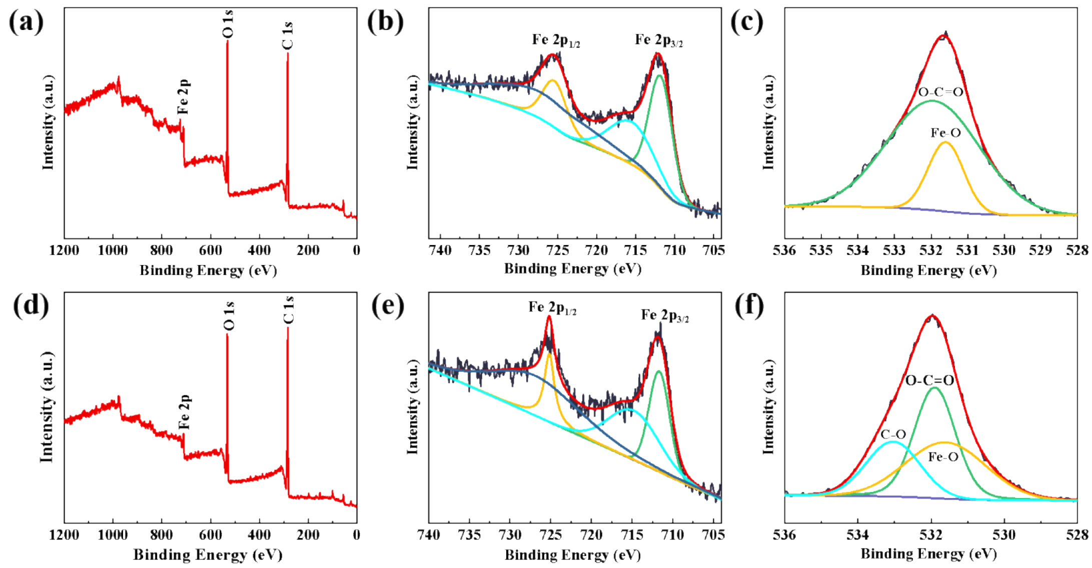

Figure S5. XPS spectra of MIL-88A: survey spectra (a), Fe 2p (b), O 1s (c), and MIL-88A/GO: survey spectra (d), Fe 2p (e), O 1s (f).

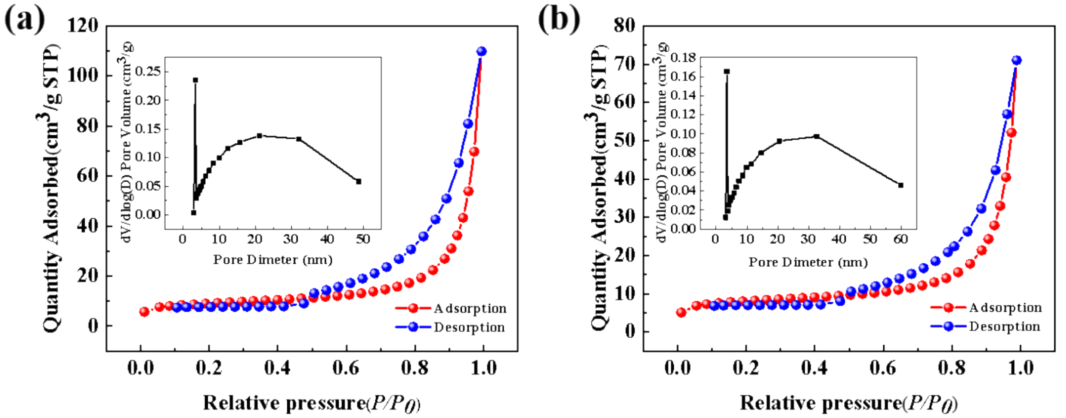

Figure S6. N_2 adsorption and desorption isotherms isotherms and pore size distribution of the FeCoNi-CH (a) and FeCoNi-CH/p-rGO (b).

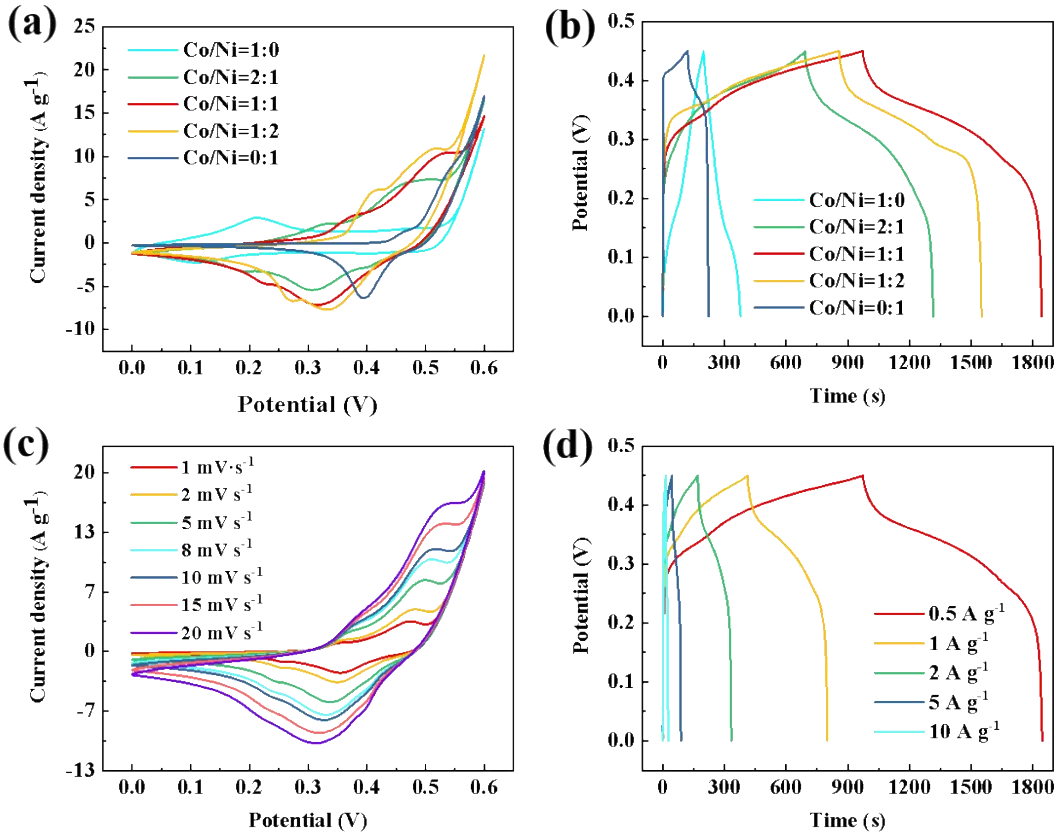

Figure S7. CV curves of FeCoNi-CH with different ratios of Co/Ni at a scan rate of 5 mV s^{-1} (a). GCD curves for FeCoNi-CH with different ratios of Co/Ni tested at a current density of 0.5 A g^{-1} (b). CV curves of FeCoNi-CH with Co/Ni ratio of 1:1 at different scan rates (c). GCD curves of FeCoNi-CH with Co/Ni ratio of 1:1 at different current densities (d).

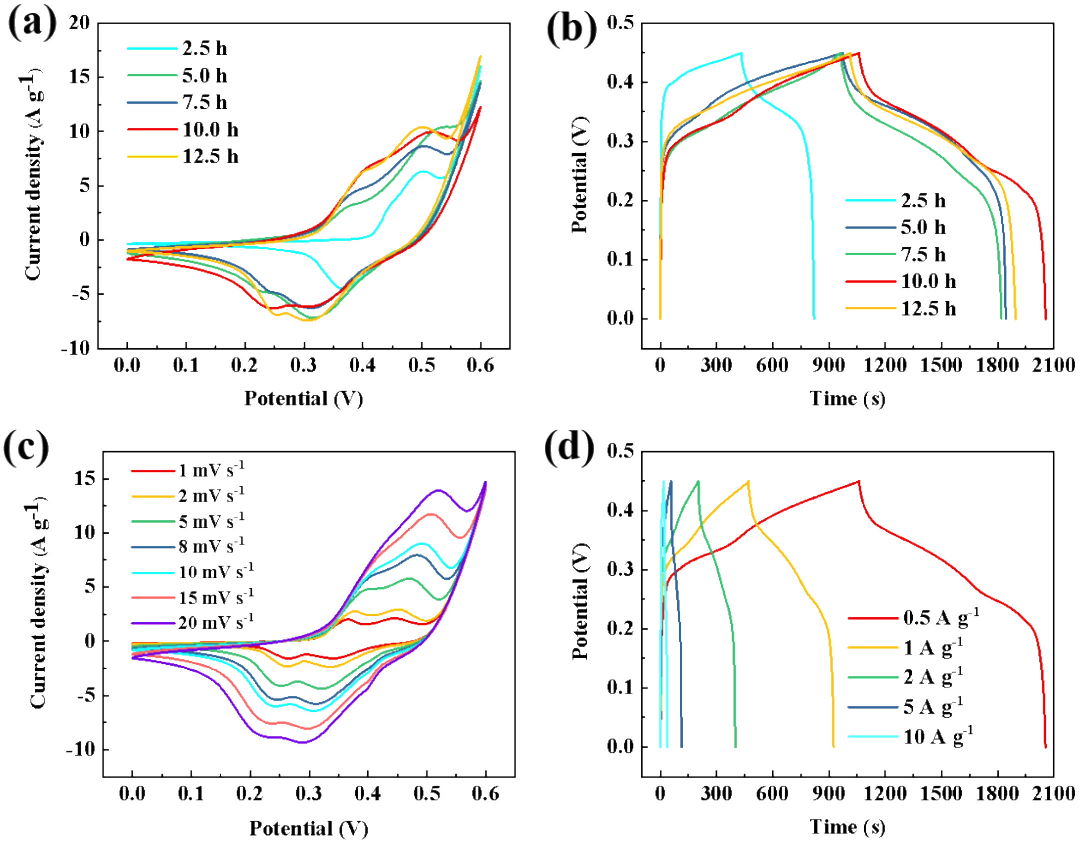

Figure S8. CV curves of FeCoNi-CH with different etching times at a scan rate of 5 mV s^{-1} (a). GCD curves for FeCoNi-CH with different etching times at a current density of 0.5 A g^{-1} (b). CV curves of FeCoNi-CH with etching time of 10 h at different scan rates (c). GCD curves of FeCoNi-CH with etching time of 10 h at different current densities (d).

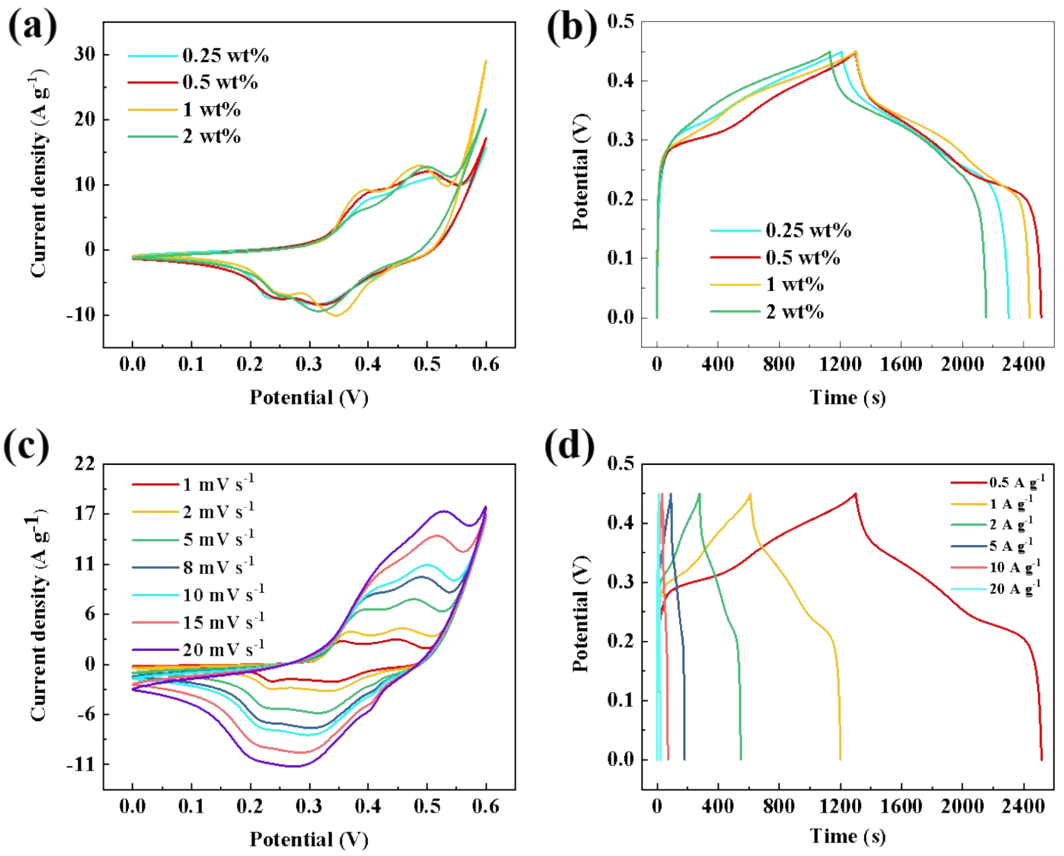

Figure S9. CV curves of FeCoNi-CH/p-rGO with different ratio of GO at a scan rate of 5 mV s^{-1} (a). GCD curves for FeCoNi-CH with different ratios of GO at a current density of 0.5 A g^{-1} (b). CV curves of FeCoNi-CH/p-rGO with GO content of 0.5 wt% at different scan rates (c). GCD curves of FeCoNi-CH/p-rGO with GO content of 0.5 wt% at different current densities (d).

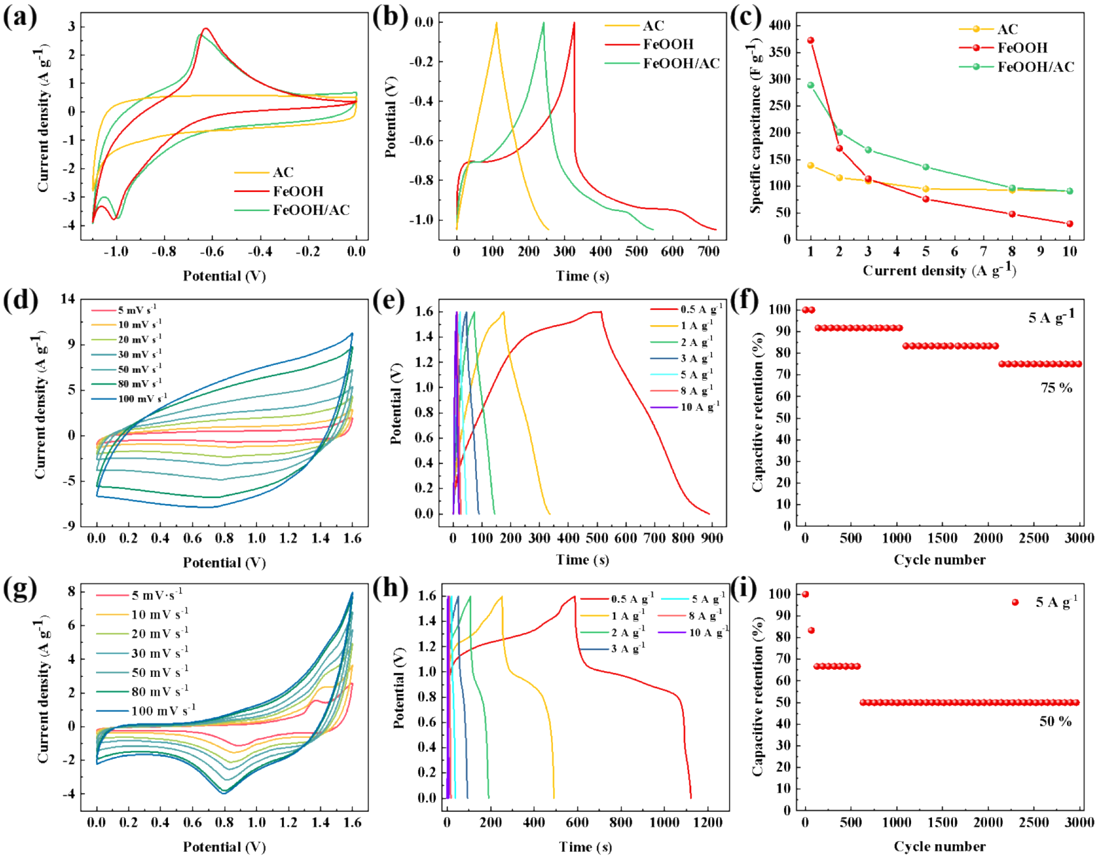

Figure S10. CV curves at a scan rate of 5 mV s^{-1} (a), GCD curves at current density of 1 A g^{-1} (b) and GCD curves at different current densities of AC, FeOOH and FeOOH/AC (c). Electrochemical performance of the FeCoNi-CH/p-rGO//AC HSC: CV curves at different scan rates (d), GCD curves at various current densities (e), cycling performance at a current density of 5 A g^{-1} (f). Electrochemical performance of the FeCoNi-CH/p-rGO//FeOOH HSC: CV curves at different scan rates (g), GCD curves at various current densities (h), cycling performance at a current density of 5 A g^{-1} (i).
